# Barriers and facilitators to dissemination of non-communicable diseases research: a mixed studies systematic review

**DOI:** 10.3389/fpubh.2024.1344907

**Published:** 2024-10-02

**Authors:** Ana Renda, Heidi Turon, Michelle Lim, Luke Wolfenden, Sam McCrabb, Seán R. O’Connor, Meghan Finch, Natasha Smith, Navdeep Goraya, Cheryce L. Harrison, Shaan Naughton, Alice Grady, Rebecca Hodder, Kathryn Reilly, Serene Yoong

**Affiliations:** ^1^School of Medicine and Public Health, University of Newcastle, Newcastle, NSW, Australia; ^2^National Centre of Implementation Science (NCOIS), University of Newcastle, Newcastle, NSW, Australia; ^3^Sydney Local Health District, Population Health, Sydney, NSW, Australia; ^4^Hunter Medical Research Institute, New Lambton Heights, NSW, Australia; ^5^Hunter New England Local Health District, Population Health, New Lambton, NSW, Australia; ^6^Monash Centre for Health Research and Implementation, School of Public Health and Preventive Medicine, Monash University, Clayton, VIC, Australia; ^7^Institute of Nursing and Health Research, Ulster University, Belfast, Ireland; ^8^Austin Health, Heidelberg, VIC, Australia; ^9^Department of Health Research Methods, Evidence, and Impact, McMaster University, Hamilton, ON, Canada; ^10^Global Centre for Preventive Health and Nutrition (GLOBE), Institute for Health Transformation, Deakin University, Geelong, VIC, Australia

**Keywords:** dissemination, public health, non-communicable diseases, implementation science, barriers and facilitators

## Abstract

**Background:**

There is a large number of research studies about the prevention of non-communicable diseases (NCD), with findings taking several years to be translated into practice. One reason for this lack of translation is a limited understanding of how to best disseminate NCD research findings to user-groups in a way that is salient and useful. An understanding of barriers and facilitators to dissemination is key to informing the development of strategies to increase dissemination. Therefore, this review aims to identify and synthesise the barriers and facilitators to dissemination of NCD research findings.

**Methods:**

A mixed studies systematic review was performed following JBI (formerly known as Joanna Briggs Institute) methodology. The search included articles from January 2000 until May 2021. We conducted a comprehensive search of bibliographic and grey literature of five databases to identify eligible studies. Studies were included if they involved end-users of public health research that were decision-makers in their setting and examined barriers/facilitators to disseminating research findings. Two pairs of reviewers mapped data from included studies against the Framework of Knowledge Translation (FKT) and used a convergent approach to synthesise the data.

**Results:**

The database search yielded 27,192 reports. Following screening and full text review, 15 studies (ten qualitative, one quantitative and four mixed methods) were included. Studies were conducted in 12 mostly high-income countries, with a total of 871 participants. We identified 12 barriers and 14 facilitators mapped to five elements of the FKT. Barriers related to: (i) the user-group (*n* = 3) such as not perceiving health as important and (ii) the dissemination strategies (*n* = 3) such as lack of understanding of content of guidelines. Several facilitators related to dissemination strategies (*n* = 5) such as using different channels of communication. Facilitators also related to the user-group (*n* = 4) such as the user-groups’ interest in health and research.

**Conclusion:**

Researchers and government organisations should consider these factors when identifying ways to disseminate research findings to decision-maker audiences. Future research should aim to build the evidence base on different strategies to overcome these barriers.

**Systematic review registration:**

The protocol of this review was deposited in Open Science Framework (https://doi.org/10.17605/OSF.IO/5QSGD).

## 1 Introduction

Non-communicable diseases (NCD) are a major public health concern, responsible for 71% of all deaths globally (1). NCD can be defined as “[…] a group of diseases linked by common risk factors, determinants, aetiologies, and pathologies, which can cause a variety of organ and organ system damage […] and are caused by the duration and dosage of exposure to anthropogenic risk factors, usually over several decades” (2, 3). In 2019, NCD accounted for approximately 1.6 billion disability-adjusted life years (DALY) lost worldwide, where one DALY equals one lost year of healthy life (4). The burden of NCD are highest among those aged 50–74 age group, with cardiovascular diseases, diabetes mellitus, chronic obstructive pulmonary disease, and various cancers being the leading causes of mortality (3). The primary modifiable risk factors contributing to the burden from NCD include physical inactivity, exposure to tobacco use, unhealthy diets and high consumption of alcohol (5–9). These risk factors can be targeted to reduce the incidence of NCD (10).

In past decades, public health research has resulted in evidence-based guidelines and plans that can inform public health policy and practice to reduce the risk of NCD by targeting their risk factors (11). Ensuring that this research evidence is used to inform public health policy is key to guarantee the benefits of research reaching the population. Governments and organisations report using different types of evidence in policy and practice decisions, however, various barriers to the use of evidence by policymakers have been identified (12). These include a lack of availability of research, lack of relevant research, poor dissemination and lack of managerial support (12, 13). As a result, newly published research or guidelines are often not translated into practice and, when they are, often taking a significant amount of time to influence policy and practice (14).

Despite guidance being available to support targeted dissemination of research, public health researchers do not disseminate research findings effectively to non-academic audiences (15). Understanding barriers to the dissemination process by public health researchers helps with better producing and disseminating NCD research. Dissemination is different from passive transfer of knowledge or *diffusion,* where the information spreads unintentionally (16). To improve research dissemination, it is important to comprehensively understand factors that promote or impede the dissemination process. Glasgow and colleagues have suggested that to enhance the integration of research into practice, barriers to dissemination need to be anticipated and be used to develop dissemination strategies (17). For the purpose of this review, we will be using the following adapted dissemination definition by Rabin and colleagues: “an active approach of spreading evidence-based *research findings* to the target audience via determined channels using planned strategies” (18).

The study of disseminating health research is not new (19); however, it is widely recognised that it is underdeveloped when compared to literature surrounding implementation science (16, 20–22). In contrast to implementation science, there is also a lack of consensus on a definition for dissemination research, limited understanding of dissemination determinants and outcomes and an almost absence of research on dissemination strategies (16, 23). One of the reasons for this is that the literature does not draw out dissemination as separate from implementation, often conflating the two. This review focuses on extending the evidence base related specifically to dissemination only (i.e., the process of getting research evidence to end-users in a way that supports practice and decisions making).

To the best of our knowledge, there is limited evidence that identifies factors that hinder or enable dissemination of public health research findings, and no previous systematic reviews synthesising the key factors that influence the dissemination of NCD research evidence specifically. Therefore, we sought to undertake a systematic review of barriers and facilitators using the Framework for Knowledge Translation (FKT) by Jacobson and colleagues as a guiding frame for synthesis (24). The FKT is a particularly useful framework describing five themes by which knowledge translation is affected: (1) the user group, (2) the issue, (3) the research, (4) the researcher-user relationship, and (5) dissemination strategies. The literature on dissemination science provides multiple frameworks and models that allow the examination of dissemination processes, agents, levels, and interactions (16, 25, 26). We opted to select the FKT for the following reasons, (a) it is dissemination only framework, and (b) comprehensively outlines the process for dissemination and the factors influencing each process (25).

## 2 Review question

This review aims to answer the following research question: What are the barriers and facilitators that disseminators (i.e., source) of research findings face when actively transferring research (i.e., dissemination) related to the prevention of NCD, from an end-user perspective.

### 2.1 Inclusion criteria

Following the JBI (formerly known as Joanna Briggs Institute) methodology, we defined and described the Population, phenomena of Interest and Context (PICo) below.

#### 2.1.1 Population

We considered studies where the dissemination of NCD research was directed to end-users of public health research that were decision-makers in their setting. We have defined end-users as: public health practitioners who are healthcare providers in the community, community members who are not health care providers but who have the authority to decide whether health programs should be implemented (i.e., school principals, regional school managers), public health researchers and academics, research funders (i.e., government, private industry, foundations, professional organisations), regulatory bodies (i.e., government departments that manage and provide recommendations or standards relevant to public health), industry members, and policymakers (i.e., government health entities responsible for overseeing, developing, implementing and evaluating public health policy and strategies). We included studies where there were different types of participants if data pertaining to participants meeting the eligibility criteria was reported separately. For example, if the sample included researchers, healthcare providers and parents, we only took into consideration data from researchers and healthcare providers. Studies targeting community members such as patients, parents, children, and the older adult were excluded as these are not considered decision-makers in their setting. The terminology defining end-users can vary among the literature and in the FKT, therefore for the purpose of this review, end-user and user-group are both used to refer to the population sample.

#### 2.1.2 Phenomena of interest

This review includes studies that explored the barriers and facilitators to the dissemination of NCD research findings by researchers or organisations responsible for disseminating research. For this study, the definition of a barrier was adapted from Bach-Mortensen et al. (27) as any factors that obstruct the dissemination of evidence-based findings and the adapted definition of a facilitator is any factor that enables the dissemination of evidence-based findings (27).

#### 2.1.3 Context

For implementation research, context “is the set of circumstances or unique factors that surround a particular implementation effort” (28). Dissemination science can be key to helping research reach NCD policymakers and practitioners, however, it has been conventionally studied conjointly with implementation science, known as dissemination and implementation science or ‘D&I’. Recent evidence suggests that dissemination is a distinct construct from implementation and therefore systematic efforts to study this process are warranted (16, 20).

#### 2.1.4 Types of studies

This review considered quantitative, qualitative and mixed methods studies, as long as they investigated barriers and facilitators to dissemination of public health research related to NCD. We excluded systematic reviews, randomised controlled trials, protocols, commentaries, editorials, comments/reviews of another paper, book reviews, narrative/literature reviews, letters to the editor, papers describing measures and conference abstracts. We included studies published in any language from January 2000, as we anticipate that the evidence surrounding dissemination is likely to have more formally emerged since then (18, 29).

## 3 Methods

This review was conducted and reported according to the JBI convergent integrated approach for mixed methods systematic reviews (30). For this review, we chose a mixed methods approach integrating data from qualitative, quantitative and mixed methods studies as this provides a more complete understanding of the type, breadth and depth of barriers and facilitators that are emerging through different research approaches such as exploratory, explanatory or convergent (31). In the latter, equal priority is given to the different types of data and results are merged (32). For example, qualitative data might add contextual information or illustrations to a numerical answer or quantitative data can provide frequency, magnitude and effects of factors regarding the existence of particular barriers and/or facilitators (32, 33). A protocol for this review has been prospectively deposited in Open Science Framework (OSF) 10.17605/OSF.IO/5QSGD.

### 3.1 Search strategy

A search strategy was developed in conjunction with a university health research librarian. The search included articles from January 2000 until May 2021.

### 3.2 Information sources

We conducted a comprehensive search of bibliographic and grey literature databases to identify eligible studies. We searched several databases including Medline, Psycinfo, and EBSCO Search Ultimate (focus on health, communications and business/marketing databases). A list of keywords for Medline and PsycInfo are found in Supplementary Data Sheet S1. Based on previous recommendations (34), the top 200 search results in Google and Google Scholar were also screened for articles and the terms *dissemination* and *public health* were used. We searched the reference lists of relevant reviews to locate additional primary studies.

### 3.3 Study selection

Search findings were uploaded to Covidence (35) and one team member removed duplicates (HT). Pairs of team members (HT, NS, SO’C, SN, EW, SMc, AR, CH, SY) screened titles and abstracts for relevance independently. Where conflict between reviewers could not be resolved, a third reviewer (i.e., a senior researcher) decided on inclusion (SY). Full text articles of relevant studies were retrieved and reviewed independently by pairs of reviewers (MF, HT, AR, NS, SY), with a third reviewer where conflicts arose (SY). Studies that met the inclusion criteria were included in the review.

### 3.4 Assessment of methodological quality

For all included studies, pairs of team members appraised them individually using JBI critical appraisal tools for qualitative, analytical cross-sectional, case controls, case reports (36) (AR, ML, NG). For mixed methods studies, we used the mixed methods appraisal tool (MMAT) (37). We included all studies regardless of their quality.

### 3.5 Data extraction

Following JBI methodology, we conducted data extraction using an adapted version of the JBI template explicitly deemed by authors and extracted by independent pairs of researchers (AR, ML, NG). We extracted the suggested fields (author, year, methodology, methods, number and characteristics of participants, aim/objective of study, phenomena of interest and setting and other context-related information) and the five elements of the FKT: the user-group, the issue, the research, the researcher-user relationship and dissemination strategies (24). These five elements are considered the *themes* of our review. We defined them and included the definitions in Supplementary Data Sheet S2. We chose the FKT as it is a dissemination-only framework, it provides construct flexibility and it addresses the complexity of knowledge dissemination across the socio-ecologic framework (25).

We then extracted verbatim qualitative and quantitative data into each of the themes. To reduce the need to interpret findings, we extracted those findings that were deemed as either barriers or facilitators to dissemination in included studies. However, there were occasions where the review team met to decide whether a factor was a barrier or facilitator when it was not explicitly reported.

Consistent with the JBI methodology, we assessed for quality of individual findings using a scale of: (1) Unequivocal (“findings accompanied by an illustration that is beyond reasonable doubt and therefore not open to challenge”), (2) Credible (“findings accompanied by an illustration lacking clear association with it and therefore open to challenge”), (3) Not supported (“when 1 nor 2 apply and when most notable findings are not supported by the data. Should not be included in synthesis to inform practice”) (see Supplementary Data Sheet S3 for not supported findings which are not included in the synthesis), and (4) not applicable—qualitized finding (see Supplementary Data Sheet S4).

### 3.6 Data transformation

After extracting data, two reviewers independently “qualitized” the quantitative evidence by converting it to a narrated version of the finding. This method was preferred above converting qualitative into quantitative findings as it is difficult to attribute a numeric value to a qualitative finding (38). As there is no step-by-step guidance to data transformation suggested by JBI, two team members (AR, ML) independently transformed each quantitative finding into a ‘qualitized’ finding, met to discuss disagreements, and consolidated the findings (Supplementary Data Sheets S5, S6).

### 3.7 Data synthesis and integration

**Figure 1:**
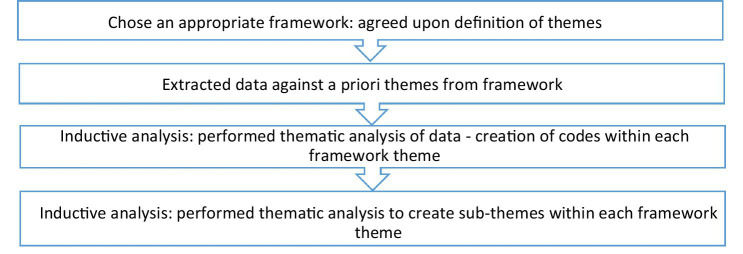
Data synthesis and integration process.

After the findings were transformed, we inductively performed thematic analysis based on suggested methodologies (39–41), where two reviewers (AR, ML) created codes of all findings per theme of the FKT independently. The team members met to discuss the coding until reaching agreement. For example, we coded the findings extracted from “the research” theme and met to discuss discrepancies. Then, we inductively converted the codes into sub-themes through thematic analysis (40, 42). Finally, we discussed the refining of sub-themes with members of the team until reaching agreement. Figure 1 illustrates this process. Themes, sub-themes and codes were summarised through a narrative description and in a table format in the results section.

## 4 Results

### 4.1 Study inclusion

#### 4.1.1 Descriptive results

**Figure 2:**
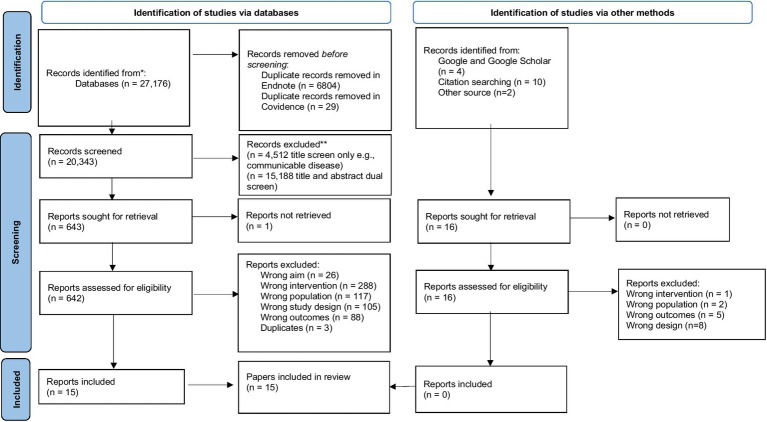
PRISMA flowchart. From: Page et al. (43).

The initial search yielded 27,192 results. Of these, 20,343 were retained after removing duplicates. After title and abstract screening, 658 reports were retained for full-text screening and those that did not meet the criteria were excluded due to wrong study design (*n* = 105), wrong population (*n* = 117), wrong outcomes (*n* = 88), wrong aim (*n* = 26), wrong intervention (*n* = 288), duplicates (*n* = 3), full-text not found (*n* = 1). Further, 1 report was not retrieved as its full-text version could not be accessed despite repeated attempts. We included a total of 15 full-text articles as shown in the Preferred Reporting Items for Systematic Reviews and Meta-Analyses (PRISMA) flow diagram in Figure 2 (43). Table 1 shows the characteristics of studies included.

**Table 1:** Characteristics of included studies.

Author, year	Methodology, methods	Methods	# of participants	Characteristics of participants	Aim/objective of study	Phenomena of interest	Setting and other context-related information
**Quantitative**							
Brownson, 2007 (34)	Cross-sectional, survey	Survey	49	Program manager or administrator, health educator, program planner, division or bureau head, other	to describe the relative importance of various factors in state-level decision making, to categorize the stage of adoption for evidence-based physical activity interventions in state and territorial public health departments, to characterize training needs to enhance awareness, understanding, and use of the Community Guide, to examine the associations between a variety of factors and the stage of dissemination of evidence-based strategies to promote physical activity.	This study was undertaken to better understand the dissemination of physical activity interventions across the United States of America (USA), focusing particularly on the evidence-based reviews in the Community Guide.	USA—all state health departments, Guam, and the Virgin Islands.
**Mixed-methods**							
Ritchie, 2021 (46)	Cross sectional and qualitative thematic analysis	Interviews	28	Health professionals	To make a step towards addressing the lack of knowledge about the impact of the European Code Against Cancer (ECAC) by investigating public awareness of it in several European countries and interrogating factors affecting its promotion and dissemination.	For over three decades, the ECAC has been used by health authorities and civil society organisations in Europe as a key tool to improve health literacy about cancer prevention. Yet, despite this adoption of the ECAC, the evaluation of its impact has been limited to several small-scale investigations with limited scope. The absence of a broader evaluation means that the extent to which the ECAC can produce changes in knowledge, attitudes, and behaviours towards cancer prevention at the individual level is largely unknown.	8 European countries (Finland, France, Hungary, Ireland, Poland, Portugal, Spain and UK).
Mattran, 2013 (33)	Cross-sectional and qualitative content analysis	Survey	135	Public health professionals, state or local government employees	To evaluate awareness of, access to, and use of the State Indicator Report on Physical Activity (SIRPA) and its accompanying resources (national and state-specific action guides, data-to-action PowerPoint presentation, data tables with [chief investigators], and press release template). The evaluation also addresses how well the materials have been disseminated to target audiences and how the value and/or quality of available content could be improved for future publications.	To aid in facilitating the goal of increasing physical activity among all Americans, the State Indicator Report on Physical Activity, 2010 (SIRPA) was initially released in May 2010 by the Centers for Disease Control and Prevention (CDC) on the CDC Website. The report provides information on physical activity behaviour, policy, and environmental supports for each state.	USA
McBride, 2007 (40)	Cross-sectional, process evaluation	Survey and interviews	35	Teachers	This study focused upon evaluating the behavioural impact and the results indicated that wider dissemination would be of value	This paper discusses the implementation and results of the formal National School Health and Alcohol Harm Reduction (SHAHRP) Dissemination Project.	Australia: South Australia (three sectors); Australian Capital Territory (three sectors); Tasmania (three sectors); and the Wodonga District in Victoria.
Williams, 2016 (37)	Cross-sectional	Survey	212	Directors, health professionals	To describe the design, development, and evaluation of an informational packet created to disseminate information about motivational interviewing to healthcare professionals at 92 community health organizations.	Printed educational materials (PEMs), such as peer-reviewed journal publications, treatment guidelines, monographs, and leaflets, are one of the most common dissemination strategies for communicating information about evidence-based practices (EBP) to healthcare professionals. Given that PEMs usually cost less than active dissemination strategies, they may be an effective option for other purposes, such as increasing healthcare professionals’ awareness of an EBP earlier in the EBP adoption process.	USA
**Qualitative**							
Dagenais, 2015 (38)	Multiple-case study approach	Documentation, daily log, summary report, survey, and interview	4	Planners and program developers, coordinators of children’s health promotion programs	To revisit the results of the qualitative evaluation. It looks critically at the theoretical foundations of the Knowledge Broker (KB) interventions in light of two conceptual models: (1) the dissemination model underlying the KB interventions used in the Canadian trial and (2) a systemic KB model developed later.	The aim of that 1-year project, funded by the Canadian Institutes of Health Research, was to foster “instrumental use” of research evidence by public health decision-makers for planning health promotion programs addressing healthy body weight in children. In parallel, a qualitative study was undertaken in the province of Quebec to collect more information on the implementation process, conditions for effectiveness, and perceived effects of the KB strategies used as interventions	Canadian project, both French- and English-speaking regions.
Faulkner, 2016 (30)	Not stated (constant comparative method)	Focus groups	104	Teachers, paediatricians, and qualified exercise professionals	To explore stakeholder (parents, teachers, exercise professionals, paediatricians, and youth) perceptions of the Movement Guidelines and identify their acceptability, perceived barriers to implementation, and recommended methods and messengers of dissemination.	Physical inactivity and obesity among children and youth are significant public health concerns today. These are largely due to low engagement in moderate-to vigorous-intensity physical activity (MVPA) and increasing time spent. As described in this special issue, there is growing evidence that light-intensity physical activity (LPA) is also important for the health of children and youth. There is also growing recognition of the need to examine the continuum of movement behaviours and to further understand the interactions between sleep, sedentary behaviour, and light-to moderate–vigorous-intensity physical activity. This recognition stimulated the development of the Canadian 24-Hour Movement Guidelines for Children and Youth (“Movement Guidelines”).	October 2015 to January 2016 in Toronto, Hamilton, Ottawa, and Vancouver, Canada.
Mitton, 2009 (35)	Grounded theory (constant comparative analysis)	Interviews	15	Researchers, decision makers and clinicians	The objective of the knowledge transfer and exchange (KTE) project was to examine the state of the KTE literature and conduct a series of key informant interviews in order to design a KTE strategy for the Alberta Depression Initiative (ADI) projects. In this paper we outline how the views expressed through the interviews directly informed the design of the KTE strategy.	Depressive disorders are highly prevalent and of significant societal burden. In fall 2004, the ‘Alberta Depression Initiative’ research program was formed with a mission to enhance the mental health of the Alberta population. A key expectation of the ADI is that research findings will be effectively translated to appropriate research users. To help ensure this, one of the initiatives funded through the ADI focused specifically on KTE. A fourth project—reported here—that focused specifically on researching KTE strategies for the core projects was also funded. The relevance of KTE has grown in recent years as funders demand greater impact for research dollars, researchers seek to have their findings impact decision making directly, and decision makers desire greater defensibility and accountability in making difficult decisions in complex environments.	Alberta, Canada. September and December 2006.
Brown, 2017 (39)	Interpretive	Interviews	30	Researchers, school staff, and public health stakeholders	Explores factors that influenced COMPASS knowledge exchange activities, from the perspective of researchers and knowledge users (i.e., school and public health stakeholders).	The research is part of a larger convergent parallel mixed-methods exploring the implementation and outcomes of the COMPASS knowledge exchange strategies. The COMPASS Study is an ongoing longitudinal study (2012–2021) of student health behaviours and secondary school environments in Ontario and Alberta, Canada.	Ontario and Alberta, Canada
Evenson, 2013 (31)	Interpretive	Interviews	27	Public health practitioners	To determine early awareness, dissemination, uses, challenges, and future recommendations of the National Physical Activity Plan (NPAP) and the companion implementation plan.	Guidance is lacking on best practices for dissemination of evidence-based physical activity interventions, particularly with a focus on policy. Moreover, dissemination of national plans is often not evaluated. The United States NPAP is the first national-level plan in this country to focus exclusively on physical activity and follows the physical activity guidelines released in 2008 by the US Department of Health and Human Services.	USA
Haynes, 2018 (45)	Interpretive: phenomenology theory and understanding	Interviews	21	Policymakers, practitioners, or both	To examine the nature of the integrated knowledge translation (iKT) activities/collaborations that facilitated the partnership; the enablers and barriers; the iKT approaches that were adopted; and the impact the partnership had on the intermediaries’ dissemination of the research findings.	Three workplace-based iKT projects were, respectively, named Sun Safety at Work Canada, the Burden of Occupational Cancer, and Completing the Picture. The common objective of the projects was examining ways to prevent or reduce occupational exposure to carcinogens. The studies were multidisciplinary and had researchers from multiple research centres.	Canada
Waqa, 2013 (42)	Interpretive (content and thematic analyses)	Interviews	35	Government and non-government organizations.	The research question for The Translational Research on Obesity Prevention in Communities (TROPIC) the TROPIC project was: Can a knowledge-brokering approach advance evidence-informed policy development to improve eating and physical activity environments in Fiji? In this study, the primary objective of the knowledge-brokering team was to exchange information with participants and participating organisations.	The TROPIC project investigated the effect of knowledge-brokering approaches on the uptake of evidence from OPIC and other sources to inform obesity-related policy in six organizations in Fiji. In line with Lavis et al., one of the main targeted outcomes of TROPIC was to utilize research evidence in the development of policy briefs, leading to more effective policy decisions and practices and, subsequently, improved health outcomes.	Fiji—TROPIC was a three year (June 2009 to October 2012) project funded by an AusAID Australian Development Research Award grant.
Riazi, 2017 (32)	Not reported	Interviews + focus groups	10	Family physician, paediatricians, ECEs in administrative positions, physical activity communicators, and researchers	To replicate the process described by Faulkner and colleagues and had the following objectives: (1) to explore stakeholder (experts in paediatric and family medicine, physical activity knowledge translation, and child care) and end user (parents and child care professionals) perceptions of the Movement Guidelines, and (2) to identify their acceptability, perceived barriers to implementation, and recommended methods and credible messengers of dissemination.	In June 2016, the first 24-Hour Movement Guidelines for Children and Youth (5–17 years) were released in Canada. These novel guidelines encompassed three movement behaviours: physical activity (light, moderate, and vigorous); sleep; and sedentary behaviours within a 24-h period. The current study, was conducted concurrently with the development of the Canadian 24-Hour Movement Guidelines for the Early Years (0–4 years).	Canada (British Columbia, Alberta, Ontario and Quebec)
Boydell, 2008 (36)	Interpretive (social construction perspective)	Focus groups interviews	74	Service providers	To: (a) identify the knowledge translation needs of rural communities; and (b) ascertain the best methods for meeting those needs.	The health problems confronting rural Canada are serious, complex, dynamic, and, to some extent, uniquely fashioned by characteristics inherent to rural life and geography. But rural health research has been a victim of “benign neglect.” Because of the lack of local services, rural children with health problems are often placed in residential care facilities outside of their communities.	Canada [Five rural communities and the regions: Fort Frances (Rainy River District), Moosonee (James Bay area), Kirkland Lake (District of Timiskaming), Smiths Falls (Lanark County), and Clinton (HuronPerth County)].
Hoelscher, 2001 (41)	Case study	Unclear	Unclear	Researchers themselves	Describe a case study of the dissemination of the Child and Adolescent Trial for Cardiovascular Health (CATCH) program, in which the theoretical underpinnings, strategies used, and lessons learned in dissemination efforts are outlined.	CATCH: A multi-component, multi year coordinated school health promotion program designed to decrease fat, saturated fat, and sodium in children’s diet, increase physical activity and prevent tobacco use.	Texas, USA

There was a total of 10 qualitative (five interpretive, three grounded theory, one multiple case approach and one case study), one quantitative (one cross-sectional) and four mixed-methods studies. They were conducted in Canada (*n* = 7), United States (*n* = 5), Europe (*n* = 1), Fiji (*n* = 1) and Australia (*n* = 1). A total of 871 participants were included in the 15 studies. Studies included physical activity (*n* = 5) (44–48), mental health (*n* = 3) (49–51), occupational cancer prevention (*n* = 1) (52), multicomponent aiming to prevent cancer (*n* = 1) (53), obesity (*n* = 1) (54), and alcohol and drug abuse prevention (*n* = 2) (55, 56); and multicomponent promoting healthy behaviours (*n* = 2) (57, 58). One study was published in 2001 (56), 2008 (50), 2009 (49), 2015 (57), 2017 (46), 2018 (58), and 2019 (52), two in 2007 (48, 55) and in 2016 (44, 51), and three in 2013 (45, 47, 54).

### 4.2 Methodological quality

We assessed the methodological quality of 10 qualitative articles using the JBI critical appraisal tool for qualitative research (59). All studies addressed the congruity of the research methodology and the research question, and 90% of studies addressed the congruity of the research methodology with the methods to collect data, the representation and analysis of data, and the interpretation of results. However, seven out of ten studies did not clearly state their philosophical perspective and seven others did not clearly declare their cultural or theoretical orientation. It was therefore difficult to understand the authors’ philosophical, cultural and theoretical perspective in each study and how this could affect interpretation of findings (59). The quality of the case study could not be appraised on all items on checklist due to the reporting style (56). The JBI critical appraisal tool was used to assess the cross-sectional study (60). This study met five out of the eight criteria, however, did not meet criteria for reporting on confounding factors and validity of measures (see Tables 2, 3). Four mixed-methods studies were appraised using the MMAT, three achieved high quality in the integration of both methodologies. All studies showed high quality in conducting the qualitative section of the studies. However, in the quantitative section of three studies, authors did not report on the representativeness of the target population (see Table 4).

**Table 2:** Assessment of methodological quality of qualitative studies using the JBI critical appraisal tool.

	Critical appraisal criteria questions									
Author, year	Q1	Q2	Q3	Q4	Q5	Q6	Q7	Q8	Q9	Q10
Brown, 2018 (39)	U	Y	Y	Y	Y	Y	U	Y	Y	Y
Faulkner, 2016 (30)	U	Y	Y	Y	Y	N	Y	Y	Y	Y
Dagenais, 2015 (38)	Y	Y	Y	Y	Y	N	Y	U	U	Y
Mitton, 2009 (35)	U	Y	Y	Y	Y	N	N	Y	Y	Y
Evenson, 2013 (31)	N	Y	Y	Y	Y	N	U	Y	Y	Y
Haynes, 2019 (45)	Y	Y	Y	Y	Y	U	Y	Y	Y	Y
Riazi, 2017 (32)	N	Y	Y	Y	Y	Y	Y	Y	Y	Y
Hoelscher, 2001 (41)	NA	Y	NA	NA	NA	NA	NA	NA	U	U
Waqa, 2013 (42)	U	Y	Y	Y	Y	N	Y	Y	Y	Y
Boydell, 2008 (36)	Y	Y	Y	Y	Y	N	Y	Y	Y	Y

Q = question. Q1: Is there congruity between the stated philosophical perspective and the research methodology?—Q2: Is there congruity between the research methodology and the research question or objectives?—Q3: Is there congruity between the research methodology and the methods used to collect data?—Q4: Is there congruity between the research methodology and the representation and analysis of data?—Q5: Is there congruity between the research methodology and the interpretation of results?—Q6: Is there a statement locating the researcher culturally or theoretically?—Q7: Is the influence of the researcher on the research, and vice-versa, addressed?—Q8: Are participants, and their voices, adequately represented?—Q9: Is the research ethical according to current criteria or, for recent studies, and is there evidence of ethical approval by an appropriate body?—Q10: Do the conclusions drawn in the research report flow from the analysis, or interpretation, of the data? Y = yes meets criteria. N = no does not meet criteria. U = unclear or cannot determine if meets criteria. NA = not applicable.

**Table 3:** Assessment of methodological quality of cross-sectional studies using the JBI critical appraisal tool.

	Critical appraisal criteria questions							
Author, year	Q1	Q2	Q3	Q4	Q5	Q6	Q7	Q8
Brownson, 2007 (34)	Y	Y	Y	Y	N	N	N	Y

Q = question. Q1: Were the criteria for inclusion in the sample clearly defined?—Q2: Were the study subjects and the setting described in detail?cQ3: Was the exposure measured in a valid and reliable way?—Q4: Were objective, standard criteria used for measurement of the condition?—Q5: Were confounding factors identified?—Q6: Were strategies to deal with confounding factors stated?—Q7: Were the outcomes measured in a valid and reliable way?—Q8: Was appropriate statistical analysis used?—Y = yes meets criteria. N = no does not meet criteria.

**Table 4:** Assessment of methodological quality of mixed methods studies using the MMAT.

	Screening questions		Methodological quality criteria														
Author, year	S1	S2	Q1.1	Q1.2	Q1.3	Q1.4	Q1.5	Q4.1	Q4.2	Q4.3	Q4.4	Q4.5	Q5.1	Q5.2	Q5.3	Q5.4	Q5.5
Ritchie, 2021 (1)	Y	Y	Y	Y	Y	N	Y	Y	Y	Y	Y	Y	Y	N	N	N	N
Mattran, 2010 (2)	Y	Y	Y	Y	Y	N	Y	Y	CT	Y	CT	Y	N	Y	Y	Y	N
Williams, 2016 (3)	Y	Y	Y	Y	Y	Y	Y	Y	N	CT	Y	Y	N	Y	Y	Y	CT
McBride, 2007 (4)	Y	Y	Y	Y	Y	Y	Y	Y	N	Y	CT	Y	Y	Y	Y	Y	Y

S = screening question (“responding ‘No’ or ‘Cannot tell’ to one or both questions might indicate that the paper is not an empirical study, and thus cannot be appraised using the MMAT”) (5). S1: Are there clear research questions?—S2: Do the collected data allow to address the research questions?—Q = question. Q 1.1. Is the qualitative approach appropriate to answer the research question?—Q 1.2. Are the qualitative data collection methods adequate to address the research question?––Q 1.3. Are the findings adequately derived from the data?—Q 1.4. Is the interpretation of results sufficiently substantiated by data?—Q 1.5. Is there coherence between qualitative data sources, collection, analysis and interpretation?—Q 4.1. Is the sampling strategy relevant to address the research question?—Q 4.2. Is the sample representative of the target population?—Q 4.3. Are the measurements appropriate?—Q 4.4. Is the risk of nonresponse bias low?—Q 4.5. Is the statistical analysis appropriate to answer the research question?—Q 5.1. Is there an adequate rationale for using a mixed methods design to address the research question?—Q 5.2. Are the different components of the study effectively integrated to answer the research question?—Q 5.3. Are the outputs of the integration of qualitative and quantitative components adequately interpreted?—Q 5.4. Are divergences and inconsistencies between quantitative and qualitative results adequately addressed?—Q 5.5. Do the different components of the study adhere to the quality criteria of each tradition of the methods involved?—Y = yes meets criteria. N = no does not meet criteria. CT = cannot tell.

### 4.3 Findings of the review

Following the synthesis, we identified 12 barriers and 14 facilitators mapped to five elements of the FKT (see Tables 5, 6 for summary of findings. See Supplementary Data Sheet S2 for definitions). The themes with the most synthesised barriers were ‘user-group’ and ‘dissemination strategies’. The ‘the user-group’ theme included four studies describing three barriers and ‘dissemination strategies’ included six studies describing three barriers. The theme with the most synthesised facilitators was ‘dissemination strategies’, had six studies identifying three facilitators. The ‘research’ theme included two barriers synthesised from four studies and two facilitators synthesised from four studies. The ‘researchers-user relationship’ theme has two barriers synthesised from two studies and two facilitators synthesised from two studies. Lastly, two barriers were synthesised from two studies and one facilitator from one study that was mapped to ‘the issue’ theme. The remainder of the section details the review findings per element of the framework.

**Table 5:** Barriers to dissemination related to the framework for knowledge translation.

Barriers to dissemination	
Themes	Sub-themes (barriers) and codes
The user group	Lack of interest in learning about disseminated information (1, 2) *Not prioritising health.* *Low participation in dissemination activity.* Perceived low value of disseminated information by end-users (2, 3) *Perceiving guidelines as unnecessary.* *Not prioritising health.* Organisational and individual limited capacity for receiving information (2, 4) *Organisation’s limited absorptive capacity.* *A champion is insufficient for dissemination.* *Lacking knowledge to interpret evidence.* *Staff turn-over.*
The issue	Perceived difficulty to link research to policy (4) *Difficulty of evidence reaching policy.* Lack of communication skills of researchers (2) *Researchers’ lack of clarity in setting tasks and roles for knowledge brokers.* *Perceived difficulty of researchers to communicate with uninterested end-users.*
The research	Discrepancies in perceived importance of the content of disseminated information (3, 5) *Content of guidelines does not include behaviour of interest.* *Perceived important risk factors by end-users are missing from the guide.* *Giving the same importance to all messages included in guide.* Including complex and incompatible content within disseminated information (3, 6, 7) *End-users’ lack of understanding about content of guidelines.* *End-users are confused with technical physical activity terms and proportion of recommended physical activity levels of guidelines.* *Lack of compatibility between information disseminated and government goals and recommendations.*
The researcher—user relationship	Discrepancies in priorities and roles between members of partnerships (8) *Partnership lacking leadership.* *Organisation’s priorities weight more over potential members of partnership.* *Lacking common priorities.* Lack of awareness of previous partnership between researcher and practitioners (2) *Lacking awareness of existent partnerships.*
Dissemination strategies	Inability to address the targeted audience (3, 5, 7, 8) *Specialists cannot reach a broad audience.* *Inability to identify the targeted audience.* *Addressing the general population with a “one-size-fits-all” approach.* *Uncertain dissemination timing.* Mismanagement of knowledge brokers (2) *Undervaluing knowledge brokering outputs.* *Lacking consistent knowledge brokering procedures.* *Inconsistent expectations for the knowledge broker.* Inadaptation of the content to its audience (5, 9) Standardising messages in plan. *Information was too technical, short and not easy to read.* *Plan disseminated is too lengthy.* *Omitting content perceived as important.*

**Table 6:** Facilitators of dissemination related to the framework for knowledge translation.

Facilitators of dissemination	
Themes	Sub-themes (facilitators) and codes
The user group	Using a preferred mode of dissemination such as onsite workshops, telephone help lines or having an expert to answer questions and interest in participating in training would facilitate dissemination (1, 2). *Telephone help line as second preferred mode of dissemination.* *Expert to answer questions as third preferred mode of dissemination.* *Grant writing as fourth preferred mode of dissemination.* *Using a CD-ROM as fifth preferred mode of training.* *Onsite workshop as preferred mode of dissemination.* *Interest in participating in training.* *Participating in training.* *Continuing sharing the information after training.* End-users valuing health and research (2–4). *Valuing health.* *Valuing health and research.* *Organisation prioritises health or research.* *Valuing research.* Having a champion, leader or knowledge broker to communicate information disseminated (5, 6). *Identifying champions/leaders.* *Playing an influential role.* *Accepting knowledge broker support.* Prior awareness of information disseminated in forms of guidelines or plans (2). *Prior awareness of guide.* *Prior awareness of guide through website.*
The issue	Organisation prioritises health (2) *Promoting physical activity is high priority for health department therefore more open to receiving information.*
The research	Quality of research included (7, 8). *Including comprehensive content.* *Including robust evidence.* *Consistently including content.* Facilitating comprehension of information included (6, 8, 9). *Presentation of information easier to access.* *Including relevant content.* *Clarity, conciseness, and systematic presentation of guidelines.* *Highlighting important content.* *Clarity of content.*
The researcher—user relationship	A relationship based on respect and trust (4, 5). *Having an opened and respectful interaction between partners.* *Having credible and independent project leaders.* *Mutual respect and communication key for a successful partnership.* *Having frequent interaction.* *Having close engagement, trust and joint decision-making.* *Conducting participatory research.* *Respect for the knowledge and skills of partners.* An existent long-term relationship where both parties can benefit from (4, 5). *Working with an established relationship.* *Existing long-term relationship.* *Parties benefiting from partnership.*
Dissemination strategies	Choosing a preferred, appropriate, and existent channel for dissemination (1, 5, 6, 10–12) *Interacting face-to-face.* *Mobile apps as a preferred channel in medical setting.* *PDF files helping raise awareness.* *Email subscription helping raise awareness.* *Link to PDF files helping to raise awareness.* *Institution contacts helping raise awareness.* *Doctor or allied health professional helped raise awareness among very few participants.* *Workshops as the preferred dissemination channel.* *Disseminating information through existing and appropriate structures.* *Choosing appropriate settings to disseminate information.* *Continuing diffusion of information after training.* Having a supportive and approachable knowledge broker (3, 10, 13) *Presence of knowledge broker.* *Approachable knowledge brokers.* *Supportive and present knowledge brokers.* *Using a knowledge broker to adapt information disseminated.* *Knowledge brokers working as a team.* Creating networks including key stakeholders (1, 5) *Building communities of practice.* *Creating networks to increase connections between stakeholders.* *Including key stakeholders in the network.* Purposely formatting the content to resonate with audience (4, 6, 9) *Relatable story-telling.* *Including a wholistic approach to present information.* *Information disseminated was about right.* *Adapting resources to audience.* Strategically developing dissemination plan including key stakeholders that can endorse the information disseminated (8, 13) *Developing plans to transfer information with key stakeholders.* *Having a list of stakeholders to disseminate information.* *Endorsement and coordination by recognised organisations.*

#### 4.3.1 The user-group

The user groups targeted are outlined in Table 1 and included school principals (i.e., headmaster, school director), program managers and health professionals. Within the user-group framework theme, three sub-themes emerged as barriers. This is centred around the lack of perceived value of evidence-based guidelines. For example, one participant said *“I do not need these guidelines. I’ve been doing this in my whole career. This is not rocket science, this to me is common sense”* (44). Additionally, some user-groups may not view health as a priority for their particular setting (e.g., schools) (58). Limited capacity and inconsistency of staff (i.e., staff turnover) also hinders dissemination as information may not reach the appropriate user-groups (58).

A factor that facilitated dissemination under the user-group theme was preference for different types of communication channels (e.g., workshops or face-to-face communication). Some studies found that participants value health information as they are willing to accept health related research. Having a champion, leader or knowledge broker also facilitates dissemination (58). For example, in one study a participant reported that *“you need a specific individual identified as your dissemination manager and that individual helps working with the researchers all the way through from the start to the end of the project”* (49). A knowledge broker can be considered as “‘knowledge managers’, ‘linkage agents’, and ‘capacity builders’” (61).

#### 4.3.2 The issue

Two barriers emerged within this theme. One study reported a barrier related to the perceived difficulty of research reaching policy. For example, one participant noted: *“Less is linked back to policy… I think [research information] gets lost and stays at the clinical or at the scientific level. And they do a good job, moving that information around at that tier. It has a very hard time coming through the glass ceiling though, into the policy world”* (participant 7) (49). Another study reported the lack of communication skills of researchers.

Only facilitator identified within this theme was that if the organisation prioritises health, it increases the possibilities to improve the dissemination process.

#### 4.3.3 The research

Barriers related to the research were most frequently explored at the end of the dissemination phase. Participants reported that there was missing content that they perceived as important, or that the messages included in plan were considered with the same degree of importance which hinders dissemination. Also, the information disseminated included complex and incompatible content. For example, a participant noted that *“there seems to be a disconnect with maybe some of the federal policy recommendations and the Physical Activity Plan…. I think that the other federal organizations could do a better job of supporting that at the national level”* (45).

Participants reported that the presentation of the research, guidelines or plans was important, citing the comprehensiveness, conciseness or the clarity of the content serve as facilitators. For example, a paediatrician highlights that guidelines need to include all health habits, including key components of the message: *“I think it’s important to balance both sides of the equation … If you do not sleep, well you do not have as much energy to expend or to conduct the activities of your day. I think you are less likely to engage in physical activity. We know that there are links between sedentary behaviour, sleep, and obesity risk as well. So a lot of the outcomes that you are targeting with this [guideline] are going to be affected by sleep as well. So it all kind of fits together as one big puzzle”* (44).

#### 4.3.4 The researcher-user relationship

Two subthemes were identified as barriers related to the researcher-user relationship. Discrepancies in priorities and roles could impact the relationship between researchers and practitioners. For example, a practitioner reported: *“It is more like oil and water. It can exist in the same bucket but you never fully integrate.” “There was definitely a bit of a bump, bumping along you know, when you realized that you do not share a lot of the same vocabulary and you do not share a lot of the same working priorities and all those kind of things”* (52). Also, they reported a lack of awareness of previous partnership between researcher and practitioners.

Two subthemes were identified as facilitators. Having an established relationship based on respect and trust helps dissemination. For example, “Going into [the project] we pretty much had established the relationship in my view. I was very familiar with your work, with the type of people that you were, your passion for it… Of course we are going to at every opportunity partner with you guys” (p270) (52). Also, having a partnership provides mutual benefits to disseminate information (54). Lastly, the elements of participatory research might facilitate dissemination. As one participant reported: *“More of the work I have done in the last five years has been involved in participatory research where the researcher knows us, knows the Centre, still has some of the distance to be able to do some of their work, but I think some of the distance in traditional research is artificial, and I think it gets in the way of some of the knowledge transfer. So I think the fact that I have colleagues that I work with and trust, and know me… has made a huge difference”* (participant 15) (49).

#### 4.3.5 Dissemination strategies

Three barriers related to dissemination strategies were found. One common barrier encountered was the inadaptation of the content to the audience. One study reported that the format of the training package was difficult to understand. For example, one participant reported, *“I got a little lost with the tables explaining the characteristics of CER [comparative effectiveness research] studies of motivational interviewing. A paragraph would have been satisfactory’. Another participant reported, ‘Characteristics of the CER studies (the tables), I’d rather just a summary of what the studies found’. The participants felt the inclusion of research evidence is important, but should be used sparingly”* (51). However, two studies reported that specific characteristics of the content of the disseminated information, e.g., guidelines, would help its dissemination.

Another barrier found was the mismanagement of knowledge brokers and their outputs. These were illustrated particularly when researchers lacked knowledge of how to use the information generated by the knowledge brokers. For example, *“We were coming up with this more or less as we went along. And it becomes an afterthought sometimes, to say, “we have got all these notes but how are we storing them, how are we presenting them to people, how are we making them user friendly?” And the answer was we were not doing a very good job of that (project manager)* (58). On the other hand, having supportive and approachable knowledge broker was a key sub-theme (50, 54, 58).

Strategically developing a dissemination plan including stakeholders that can endorse the information disseminated acts as a facilitator (50, 53). For example, *“Think the whole chain out*—*what do you really want to achieve with your message to a policy maker? You must think the whole chain through and not only be clever in putting it, making a summary on one page, or to send it in terms of guidelines, you have to think all the way up to what you want to achieve at the end. And think those steps out and take action on all of them” (participant 13). “I think first of all it needs to be something that is clearly developed. It has to have who it is aimed at, who it is targeted at, what are the goals and the objectives” (participant 12) (page 6)* (49).

*Another key finding is to choose a preferred and appropriate channel for dissemination. Face-to-face workshops, organisation contacts, word-of-mouth, media, pdf files, email subscriptions and websites were cited in* var*ious studies as means of facilitating dissemination* (47, 49, 51, 54, 55). This finding is related to the sub-theme in the user-group. Further, included studies report that using existing communication channels to disseminate information included in guidelines will increase dissemination. For example, one participant (Physical Activity Communicator) explained, “In the webinars that we do, we could leverage the new guidelines, and definitely through our communications we can start some conversations around these guidelines” (page 141) (46). Another facilitator is purposely formatting the content to resonate with audience. For example, researchers when they communicate to policy-makers have said, “research really helps inform what our policy position would be. But even when you have the research, you still have the personal story. You know, the real-life example of it. It’s really important to help when you are talking to policymakers, especially… Like when you can bring it to life by having a real story to say, ‘This is why this is really important to make this policy change” (page 271) (52).

Lastly, a key facilitator was to develop networking opportunities between researchers and practitioners by including key actors in the communication chain (49, 54). A study cited that participants were struggling to reach certain stakeholders, and thanks to networking, information can be disseminated: *“[Now] I am thankful with the networking that TROPIC started as we meet and [know] the people that we [usually struggled] to see within the Ministry and those outside the Ministry and is not a challenge any more.”*

## 5 Discussion

This systematic review is the first, to our knowledge, to provide a comprehensive summary of the barriers and facilitators that affect the dissemination of NCD prevention related research to public health decision makers. It describes these findings using the five elements of the Framework for Knowledge Translation. One of the primary findings of the review is that using end-user preferred channels for dissemination, and identifying ways to integrate these with existing dissemination pathways would facilitate the dissemination of research findings. This finding is related to both user-groups and dissemination strategies. For example, one strategy to disseminate new guidelines or research would be to include this information into physical activity forums/conferences where physical activity related information is already being communicated to the workforce (46). Consistent with a review of dissemination frameworks by Baumann and colleagues, and Brownson’s Model for Dissemination of Research, the channel or medium of communication is considered a key determinant of dissemination success (16, 22). Our review found that the user-group preferred onsite workshops, telephone help lines or having an expert to answer questions to facilitate dissemination. Despite this, evidence shows that researchers tend to predominately use publications or academic conferences to disseminate their findings (62). Future controlled research examining the impact of different dissemination channels as a dissemination strategy is needed, in light of limited existing empirical research (63). Another important finding of the review is the role of knowledge brokers or champions and noted the potential usefulness when they are integrated within the end-user agencies. Our findings show that having an influential champion/individual may be a useful dissemination strategy by being a trusted, present, supportive and approachable source of information. Knowledge brokers can support end-users to change knowledge and skills and tailor knowledge products to be relevant to end-users’ needs and values (61). As Jacobson suggests, research that is related to the end-users’ beliefs and values will resonate with them and more likely lead to the adoption of the disseminated research (24). However, knowledge brokers could be seen as a barrier if their roles are not well defined (58).

Finally, the review highlights the importance of relationships between researchers and user-groups to support dissemination success. This is consistent with research co-production and integrated knowledge translation where researchers and knowledge users work together to produce research relevant to knowledge users and enhance the sharing and use of findings (64). It is therefore unsurprising that developing and fostering trustworthy and respectful relationships between researchers and end-users may help support effective dissemination of research findings. This is supported by research that highlights the need to build personal networks, relationships and partnerships to facilitate dissemination and use end-users’ preferred communication methods (6, 20, 62, 65–67). Despite this, less than half (46%) of researchers use networking (68) and do not have a strategy to build relationships beforehand. Uphold and colleagues suggest researchers find it difficult to know how to best disseminate beyond professional conferences and publications (26% of participants) (69). There is a clear disconnection between researchers, the channels chosen, the relationship between researchers and the user-group, and the knowledge about the user-group preferred channels. Future research exploring the user-group preferred channels, and research that includes both researchers and user-groups are recommended. A suggested approach is using participatory research methods (e.g., participatory codesign or community-based participatory research) which by design involves all participants or partners (i.e., researchers, end-users, knowledge brokers) in the research process, and taking into consideration their preferences through continuous communication to facilitate dissemination of research findings (23, 70).

### 5.1 Strengths

The use of a convergent mixed methods methodology which allowed for the examination of the research question through a quantitative and qualitative lens, which is best suited to provide a better understanding of factors affecting dissemination. We undertook the review consistent with best practice approaches and included a range of study designs to better provide a thorough and a contextual understanding of the factors affecting dissemination. We attempted to reduce issues related to conducting systematic reviews of barriers and facilitators (27), by including duplicate screening, factor identification, grading the factors, and data extraction.

### 5.2 Limitations

There were a number of limitations with the review. Firstly, we encountered challenges with defining dissemination and differentiating dissemination from implementation science or from knowledge translation studies. To support selection of studies, we used a definition of dissemination that was frequently employed and widely accepted in the field. Prior to commencing screening and extraction, iterative discussions with senior researchers who are experts in the field of implementation science (SY, LW) were undertaken. This enabled the research team to develop clear criteria for distinguishing dissemination activities from implementation ones. This was used throughout the review process. We used a broad search strategy to capture all possible eligible studies, however it is possible that some studies may be missed given inconsistent terminology and indexing. Furthermore, although our focus has been solely on dissemination, there is a lack of evidence suggesting that dissemination alone improves implementation (63, 71). Secondly, although a rigorous data extraction process was undertaken, identifying barriers and facilitators proved challenging due to inconsistent reporting across studies. Several rounds of consultation within the team were conducted to reach agreement; however, certain findings may have been missed due to lack of clarity in reporting.

Lastly, studies reporting findings about public health as a broad discipline were excluded as they were outside the scope of the review. We acknowledge that the public health discipline includes communicable diseases such as Human Immunodeficiency Virus, COVID-19, influenza, and other viral or bacterial diseases, and there would have provided some insights into the barriers to dissemination. However, given the differences in the way research evidence is used and therefore disseminated between communicable and NCD (72), we sought to limit the review to focus only on prevention of NCD.

## 6 Recommendations

### 6.1 Recommendations for practice and research

Our review found several barriers and facilitators that should be considered to enhance dissemination of NCD prevention research evidence. Firstly, the use of different communication channels, identifying effective, appropriate dissemination strategies and disseminators developing meaningful relationships with the user-group is recommended to facilitate an open and ongoing dialogue between the disseminators and the user-group. To further advance the science of dissemination, consistent terminology and definitions need to be applied to future research. The review by Baumann summarised the components of dissemination frameworks to guide future research, which include recommendations for a more consistent use of a dissemination definition, strategies and determinant constructs when conducting and reporting dissemination studies (17).

Secondly, our review highlights the lack of empirical research examining the dissemination process, despite its importance (63). Future empirical studies assessing dissemination determinants and strategies are needed. Studies similar to one conducted by Tabak and colleagues, which identified factors related to researchers’ efforts to disseminate findings, are encouraged. They found that having experience in practice or policy settings, as well as being a university researcher affiliated with a Prevention Research Centre, were the strongest predictors of effective dissemination (65). Furthermore, there is a need to assess the impact of dissemination strategies when applied to different contexts and audiences to overcome reported barriers. A recent scoping review by Turon and colleagues highlight the need for experimental studies comparing different dissemination strategies for effective dissemination (63). We encourage future studies to trial the dissemination of research findings with adapted content (i.e., what it contains and how it is presented) among a targeted sample, to understand the potential effect on dissemination outcomes.

Thirdly, dissemination strategies should consider audience preferences for communication channels, information format and content, and focus on addressing identified barriers, such as the lack of researchers’ communication skills and the lack of comprehensiveness of disseminated guidelines. Importantly, our review highlighted that relationships between researchers and end-users are key to support effective dissemination. Integrated research-practice roles have been identified as a way to support the translation of research and to build strong, meaningful partnerships between researchers and practitioners (73).

Lastly, the context in which dissemination occurs is likely to significantly influence its success. Therefore, we recommend conducting research aimed at understanding the current political, policy, economic context before disseminating research findings. This could be achieved through mixed methods studies that incorporate contextual information, thus informing future dissemination efforts. This recommendation is further supported by a recent review by Escoffery and colleagues which identified a lack of consensus on the definition of context and scarce empirical evidence testing context constructs in dissemination and implementation science (74).

## 7 Conclusion

Our systematic review identified that several unique barriers exist for disseminators, regarding their relationship with user-groups, and for the user-groups alone. Despite this, there are numerous facilitators that could be considered when planning to disseminate, such as having a dissemination plan prior to disseminating, improving and adapting the content to the audience and forming a respectful and trustworthy relationship with the intended audience. Future research on this topic should aim to reduce barriers and identify dissemination strategies that will increase uptake of NCD findings.
