# Supplementary material for: Barriers and facilitators to dissemination of non-communicable diseases research: a mixed studies systematic review
Source: Front Public Health. 2024 Oct 2;12:1344907. doi: 10.3389/fpubh.2024.1344907 (PMC11479996; doi:10.3389/fpubh.2024.1344907)
Supplement: Supplementary file 1 [file Data_Sheet_1.docx]

Additional File 1

Search strategy for Medline and PsycInfo. Completed in 24/05/2021.

| 1. Public health/ or public health.tw. |
| --- |
| 2. Community Health Services/ or community health.tw. |
| 3. Health promotion/ |
| 4. health promot*.tw. |
| 5. 1 or 2 or 3 or 4 |
| 6. "Diffusion of Innovation"/ |
| 7. diffusion of innovation.tw. |
| 8. Information Dissemination/ or information dissemination.tw. |
| 9. research sharing.tw. |
| 10. research trans*.tw. |
| 11. data sharing.tw. |
| 12. data trans*.tw. |
| 13. information sharing.tw. |
| 14. information trans*.tw. |
| 15. knowledge sharing.tw. |
| 16. knowledge trans*.tw. |
| 17. Evidence-Based Medicine/ed, mt [Education, Methods] |
| 18. Information Services/sn [Statistics & Numerical Data] |
| 19. Practice Guidelines as Topic/st [Standards] |
| 20. Social Marketing/ |
| 21. Social Marketing.tw. |
| 22. academic detailing.tw. |
| 23. dissemination strateg*.tw. |
| 24. disseminat*.tw. |
| 25. Health Communication/ or health communication.tw. |
| 26. 6 or 7 or 8 or 9 or 10 or 11 or 12 or 13 or 14 or 15 or 16 or 17 or 18 or 19 or 20 or 21 or 22 or 23 or 24 or 25 |
| 27. knowledge.tw. |
| 28. Reach.tw. |
| 29. Adopt*.tw. |
| 30. research utili*.tw. |
| 31. uptake.tw. |
| 32. Health Knowledge, Attitudes, Practice/ |
| 33. motivat*.tw. |
| 34. intention*.tw. |
| 35. attitud*.tw. |
| 36. awareness.tw. |
| 37. 27 or 28 or 29 or 30 or 31 or 32 or 33 or 34 or 35 or 36 |
| 38. exp animals/ not humans.sh. |
| 39. 5 and 26 and 37 |
| 40. limit 39 to yr="2000 -Current" |
| 41. 40 not 38 |
