# Supplementary material for: Barriers and facilitators to dissemination of non-communicable diseases research: a mixed studies systematic review
Source: Front Public Health. 2024 Oct 2;12:1344907. doi: 10.3389/fpubh.2024.1344907 (PMC11479996; doi:10.3389/fpubh.2024.1344907)
Supplement: Supplementary file 2 [file Data_Sheet_2.docx]

Additional File 2

Table with definitions of the elements suggested by the Framework for Knowledge Translation. Definitions were adapted to suit the review aims.

| The user group | Barriers/Facilitators to dissemination related to the user group. The user group is defined by the participants of the study, whoever is receiving the disseminated information. It is related to the context in which the user group operates, the formal or informal structures the user group is embedded, the user group as the decision-making authority, actions or decisions the user group makes, their experience with knowledge translation and research, and their status, their educational backgrounds, the length of their tenure with the organisation and their enthusiasm for the project. Based on the Framework for Knowledge Translation. |
| --- | --- |
| The issue | Barriers and facilitators related to the researcher and the context in which the research will be disseminated. The elements of the context which the researcher needs to consider when disseminating. For example, different policy sectors with different utilisation contexts and approaches, the amount of conflict or consensus surrounding an issue, different types of issues leading/not leading user groups to use research. It could be politicians supporting cancer policies, etc. |
| The research | Refers to the research or end-of-grant KT product that is being disseminated. It could be related to the message and its relevance to the user group and the congruence (meeting users’ expectations). |
| The researcher-user relationship | Barriers/Facilitators to dissemination related to the relationship between the researcher and the participant/user group. The relationship 'include the receptivity of the user group and the degree of trust or rapport that exists between the researcher and user group'. |
| Dissemination strategies | Barriers/facilitators to dissemination related to the manner, mode and venue of information. Also, B/F related to the user group’s preferences for the amount of information and level of detail, as well as how the information can be made exciting or vivid for the user group. |
