# Supplementary material for: Barriers and facilitators to dissemination of non-communicable diseases research: a mixed studies systematic review
Source: Front Public Health. 2024 Oct 2;12:1344907. doi: 10.3389/fpubh.2024.1344907 (PMC11479996; doi:10.3389/fpubh.2024.1344907)
Supplement: Supplementary file 3 [file Data_Sheet_3.docx]

Additional File 3

Findings of studies that were rated ‘not supported’ and not included in final analysis of results. Listed per theme and author.

1. *The user-group*

| ***Not included in analysis due to low quality:*** | | |
| --- | --- | --- |
| Author | Facilitator | Barrier |
| Dagenais et al, 2015(57) | The respondents particularly appreciated the broker’s support in helping them to understand and interpret the new knowledge. Page 14 | -- |
|  | With regard to organizational characteristics, the respondents from three sites reported that they made program decisions as a team. Page 15 | -- |
| McBride et al, 2007(55) | Additionally, others had heard previously of the SHAHRP project and were motivated by this prior knowledge. Page 669 | These barriers included: the uncertainty of education sectors funding and current positions making forward planning difficult; the position of teacher training in the fourth term rather than early in the year; a pre-existing workload which precluded additional workshops; a jurisdiction too small to be able to fulfil teacher workshop commitment: illness; lack of time; no support from the Principal; and moving to different schools and regions in the following year. Page 669. |
|  | Several co-ordinators commented that the year-long commitment and the clear delineation of all parties’ involvement and commitment was a motivating factor, as was the involvement of their whole drug education team in training and teacher workshops. Page 669 | Two co-ordinators were concerned that some Principals may have not passed the details about the SHAHRP programme on to the relevant people in the school. Page 670. |
| Hoelscher et al, 2001(56) | Because school health programs have limited personal and financial support within the academic environment, ongoing support from the CATCH team provided technical resources for the practitioners as well as continuity through the CATCH diffusion network. Page 98. | Cost of a program can affect dissemination efforts - most schools in Texas have limited budgets for physical education equipment and health education materials so financial support and underwriting of program costs was and important factor for dissemination. Page 97. |
|  | -- | Dissemination efforts to classroom teachers have been less robust, probably due to the fact that the classroom teacher organizations do not have a strong health, physical activity, or nutrition emphasis and it has been difficult to identify a singular social system or organization for elementary school teachers. Page 97. |

1. *The issue*

| Not included in analysis due to low quality | | |
| --- | --- | --- |
| Author | Facilitator | Barrier |
| McBride et al, 2007(55) | When asked if the researchers at NDRI could do more to make the process of dissemination easier for their organisation, all respondents agreed that they had received a great deal of support from NDRI. One coordinator suggested that an official letter from NDRI to school Principals outlining details of the SHAHRP programme, study results and the National Dissemination Project may have increased the status of the programme, and therefore support, at the school level. Page 670. | These barriers included: the uncertainty of education sectors funding and current positions making forward planning difficult; the position of teacher training in the fourth term rather than early in the year; a pre-existing workload which precluded additional workshops; a jurisdiction too small to be able to fulfil teacher workshop commitment: illness; lack of time; no support from the Principal; and moving to different schools and regions in the following year. Page 670. |
|  | One key stakeholder noted that as a small state they are more responsive to offerings, as they do not have the capacity to develop their own resources. | Other barriers included concern about […] how the SHARHP programme linked into the state drug strategy; and how to adapt SHAHRP to schools with special needs. Page 670. |
| Hoelscher et al, 2001(56) | Compatibility of the program with current practice - In 1999, the Texas State Board of Education unanimously approved the CATCH materials as a diabetes education program that schools could use as part of their health curriculum under the Texas Education Code. Page 92. | -- |

1. *The research*

| ***Not included in analysis due to low quality:*** | | |
| --- | --- | --- |
| Author | Facilitator | Barrier |
| Riazi et al, 2017(46) | Receptivity: ‘The whole picture’ - The recommendation for trading indoor for outdoor time was also well received by participants. Some child care providers reported feeling resistance from parents when taking children outside in poor weather conditions (e.g., rain), and felt that the Movement Guidelines would help support dedicated outdoor time within their programming. Page 5. | -- |
|  | ‘The whole picture’ - The majority of stakeholders recognized the relationship and interplay between the movement behaviours as well as the importance of the individual behaviours. Many also wanted and expressed a need for such guidelines. For example, researchers felt that they could use the Movement Guidelines as measurable targets in their studies, and physical activity communicators felt that the recommendations would enhance the structure and add a level of integrity to their workshops. The guidelines provided an evidence-based resource for stakeholders in medicine and early childhood education, while also providing parents with a set of recommendations to work toward. Page 5. | -- |
| McBride et al, 2007(55) | Reasons included: the evidence-based and researched nature of the programme; a focus upon alcohol; the availability of teacher training; and funding of teacher training including teacher release payments. Page 669 | -- |
|  | Several co-ordinators commented that the year-long commitment and the clear delineation of all parties’ involvement and commitment was a motivating factor, as was the involvement of their whole drug education team in training and teacher workshops. Page 669(44) | -- |
|  | Additionally, others had heard previously of the SHAHRP project and were motivated by this prior knowledge. Page 669 | -- |
| Hoelscher et al, 2001(56) | Relative advantage - CATCH was designed using state-of-the-art intervention methods and strategies and the program demonstrated positive effects. In practice, the physical education component (CATCH PE) often is cited as the most innovative due to its usefulness, convenience, teacher satisfaction and unique packaging (a modular card system with class activities). The price was also an important aspect of the CATCH dissemination effort. Because of the funding for CATCH dissemination, a modified set of materials was available for a discounted price to Texas schools. Page 92. | -- |

1. *The researcher-user relationship*

| ***Not included in analysis due to low quality:*** | | |
| --- | --- | --- |
| Author | Facilitator | Barrier |
| McBride et al, 2007(55) | When asked if the researchers at NDRI could do more to make the process of dissemination easier for their organisation, all respondents agreed that they had received a great deal of support from NDRI. One coordinator suggested that an official letter from NDRI to school Principals outlining details of the SHAHRP programme, study results and the National Dissemination Project may have increased the status of the programme, and therefore support, at the school level (page 670) | -- |
|  | Some key stakeholders indicated that direct contact with NDRI and the professional and flexible manner of project staff was important. (page 669) | -- |
| Hoelscher et al, 2001(56) | When possible, partnerships were formed with various organizations including state health agencies, the state education agency, state and national organizations, and private health foundations. Networking with these organizations provided opportunities for funding of various components and dissemination activities, provided opportunities to present CATCH information to professional groups, and led to further networking possibilities. (Page 95) | -- |
|  | Networking efforts were focused on smaller areas: systematic targeting of smaller regions yielded better results than targeting the entire state of Texas (Page 97). | -- |

1. *Dissemination strategies:*

| **Not included in analysis due to low quality:** | | |
| --- | --- | --- |
| Author | Facilitator | Barrier |
| Dagenais et al, 2015(57) | the KB intervention appeared to have facilitated the first of the five stages of the dissemination model (i.e., knowledge). | the content of the tailored messages was not sufficiently integrated with other available sources of information to enable them to fully grasp the implications of the new knowledge. |
|  | The research summaries in French were greatly appreciated, while respondents found the original English articles more difficult to understand | |
| Faulkner et al, 2017(44) | It was noted by paediatricians that primary care providers would be more effective as a main source for disseminating the Movement Guidelines. Although stakeholders described a wide range of sources of information about the guidelines, a collaborative, multi-sectoral approach was recognized as essential. | -- |
|  | Participants described that the Movement Guidelines must first be distributed by credible sources (e.g., government and nongovernmental agencies, university, and health institutions) that have a broader public outreach. Overall, “the school” or “doctors” were seen as the most common and credible sources of information about the Movement Guidelines. | -- |
|  | pamphlets and/or posters and online media were the most commonly discussed forms of dissemination. (Page s308) | -- |
|  | teachers, qualified exercise professionals, and paediatricians advocated for educational resources such as workshops and guidebooks to elaborate on how to adopt and implement the Movement Guidelines. (Page s308) | -- |
| Riazi et al, 2017(46) | Medical settings (e.g., doctor’s offices, hospitals), child care settings (e.g., daycares, home care), community centres (e.g., libraries, community schools, early years centres), and schools (for four-year-olds) were cited across interviews and focus groups. Particular emphasis was placed on physician visits, partly because promotion of healthy behaviours (such as physical activity and adequate sleep) was viewed as the “business” of physicians, and partly due to the frequency of visits among Canadian infants and preschoolers to receive publicly funded immunizations (Page 140) | -- |
|  | Other mechanisms cited were educational programs for newcomers as a part of settlement services; government mail-outs and electronic notices (e.g., notifications about the Children’s Fitness Tax Credit); and listservs and newsletters (e.g., from daycares, community centres, health professional organizations). | -- |
|  | Non-electronic formats to disseminate the Movement Guidelines included printed pamphlets, posters, magnets, professional conference presentations and booths, peer-reviewed journal articles (and their electronic versions), and a telephone help-line. Page 141. | -- |
|  | Messengers and methods of dissemination: Settings for dissemination -There was also a sense that in order to promote uptake, there would need to be an endorsement of the guidelines from professional societies, such as the College of Family Physicians of Canada, the Canadian Paediatric Society, and the Public Health Agency of Canada. These endorsements, which would ultimately enhance the credibility of the Movement Guidelines, could take the form of logos integrated within the Movement Guidelines, or web links to the guidelines posted on the websites of professional societies and government organizations. Page 140. | -- |
| Boydell et al, 2008(50) | Reading Material (brochures, posters, newsletters): Placing brochures in locations where they can easily be picked up and read, such as in the classroom or school. Posting information in places where viewing cannot be observed by others, such as on the inside doors of washroom stalls, in elevators. Posters (particularly those listing contacts, telephone numbers for getting help) should be printed with large letters so they can be read from a distance. Information packages given at the beginning of each school year to every student so that no one is singled out. Page 59. | -- |
|  | Newspapers: Include an annual insert of all the relevant community services and resources with contact information. Run a mental health column that highlights various issues with children’s mental health (eg., in a question and answer format). Page 59. | -- |
|  | Television and Radio: Public announcement ads that provide general information about children’s mental health. Page 59. | -- |
|  | Face to Face Presentations/Meetings/Workshops: Workshops and courses for teachers offered during PD days. Workshops and courses for parents on topics that are more general and non-stigmatizing (eg., better nutrition for your children). These types of sessions might attract more parents and provide a venue to give families specific information about children’s mental health. Presentations and assemblies within schools, with invitations for parents to attend as well. Page 59 | -- |
| McBride et al, 2007(55) | -- | All key stakeholders commented that the project would have been more successful in attracting teachers to workshops if the project had run in a single calender year with a planning phase towards the end of the preceding year. (Page 669-670) |
| Williams et al, 2016(51) | Some participants reported that the font, colors, graphs, and presentation of information were what they liked most about the packet. Page 91 | -- |
|  | Participants also suggested targeting the packet for specific decision makers within the organization (e.g.,providers, policymakers, directors), and creating different versions of the packet to show how the practice of motivational interviewing may work for specific treatment populations (i.e., children, severely mentally ill, chronically ill). Page 91 | -- |
| Hoelscher et al, 2001(56) | Training sessions are crucial - The training session provided direct interpersonal channels to present the CATCH program and to make contacts for future networking. Page 98 | -- |
|  | Interpersonal channels were facilitators of dissemination. Interpersonal channels such as support of opinion leaders and networking appeared to be much more effective than print media. Page 97 | -- |
|  | Although print media was necessary, this mode of communication without interpersonal networking seemed to have little effect on program dissemination. Page 98. | -- |
|  | Not only were multiple channels targeted so that messages were triangulated back to the targeted population, but these channels had credibility and accessibility. Page 98. | -- |
|  | Budget constraints also limited the ability to conduct an effective media campaign without the support of interpersonal channels. Page 98. | -- |
|  | Networking was especially crucial because the efforts of change agents can be synergistic with the dissemination team, thus leading to increased visibility of the program without additional effort on the part of the dissemination team. Page 97. | -- |
|  | Complexity - refers to the ease of implementing an innovation. CATCH curriculum and physical education materials were designed to be 'teacher friendly' and fun. | -- |
|  | Triability refers to the ability to experiment with a portion of the innovation before adopting it. CATCH materials were available during the dissemination phase through distribution of loaner copies of the CATCH package, and through lessons and recipes available on the CATCH web site. Page 95. | -- |
|  | CATCH training sessions were personalized to the targeted audience and component and varied in length (4-6 hours) depending on the topic and time available. | -- |
|  | Physical education teachers in Texas have a strong network through professional organizations, and efforts to disseminate through these channels proved to be very effective. | -- |
