# Supplementary material for: Barriers and facilitators to dissemination of non-communicable diseases research: a mixed studies systematic review
Source: Front Public Health. 2024 Oct 2;12:1344907. doi: 10.3389/fpubh.2024.1344907 (PMC11479996; doi:10.3389/fpubh.2024.1344907)
Supplement: Supplementary file 4 [file Data_Sheet_4.docx]

Additional File 4

Findings extracted by theme with their correspondent quality level.

1. **The user-group**
2. *Barriers*

The following table lists the findings extracted from each study related to the user group, deemed as barriers with its correspondent code.

| **Author** | **Codes** | **Findings with illustration** | **Quality of findings** |
| --- | --- | --- | --- |
| Faulkner et al (44) | Perceiving guidelines as unnecessary. | Receptivity: Fitting things together like one big puzzle. One paediatrician described why the Movement Guidelines are not needed: “I don’t need these guidelines. I’ve been doing this in my whole career. This is not rocket science, this to me is common sense” (PED;M;I;E). Pg S306. | Unequivocal |
| Mitton et al (49) | A champion is insufficient for dissemination. | Respondents noted, however, that it would be insufficient to rely upon single, isolated individuals to advance KTE within decision maker organizations. "I think the grassroots people, they're all over the place, so they don't really have someone to bounce ideas off of in their own Region" (P4). "Putting a single individual into an organization does no good at all; they have to become a magnet and focal point for some new processes and even new structures in the environment if they are going to be effective at doing the knowledge transfer and exchange function" (Participant 2). (page 5) | Unequivocal |
|  | Organisation’s limited absorptive capacity.  Lacking knowledge to interpret evidence. | Organizations need certain capacities in order to take up and use research knowledge - successful KTE also depends upon the capacity of decision maker organizations to interpret, contextualize, and use research evidence. This includes dedicated and appropriately skilled personnel. "We just have not developed the personnel to do this. I mean, it is becoming better but five years ago we really didn't have anybody who specialized in transfer exchange so we are starting to see the emergence of specialists in knowledge transfer and exchange more and more, but it is very, very few of them around. So we have a human resources issue." (P2) (page 5) | Unequivocal |
| Brown et al (58) | Not prioritising health. | Additionally, researchers discussed how schools that assigned low priority to COMPASS and school health were less likely to participate in knowledge brokering: "I think some schools are excited to be part of the study and the School Health Profile that they get out of it, but don’t necessarily want too much more; whether it means they’re too busy, they have more things on their plate, it’s getting close to the end of the year. (KB3)" "Where it becomes a challenge is if, you have a principal who doesn’t necessarily see the value of advancing a health agenda, and if there’s no stakeholder, that’s kind of worse case scenario. (PI)" (page 594) | Unequivocal |
|  | Staff turn-over. | Changes in school administrators, school contacts, or knowledge brokers, and the time of year schools received their results also presented challenges: “The principal kept changing, and even now, I have a new contact that’s the fourth contact” (KB4). (page 596) | Unequivocal |
|  | Not prioritising health. | Researchers highlighted the importance of school staff, especially the school administrator, valuing student health. “And almost always, it had to be an administrator. Because at times we’d get a phys. ed. teacher that was really excited, but it didn’t go anywhere because their administrator was kind of the gatekeeper to change” (KB2). (page 594) | Unequivocal |
| Evenson et al (45) | Users unfamiliar with information disseminated. | Awareness of the NPAP: All but two respondents interviewed were aware of the NPAP. One non-aware respondent stated, “I know about the physical activity guidelines, and I wonder if they were one and the same.” The other respondent not aware of the NPAP stated, “I don’t know that plan. So I haven’t really read up on it to share it. All we do is we come up with our own plan and we share it.” (Pg 2-3). | Unequivocal |
| Riazi et al (46) | Users unfamiliar with information disseminated. | However, the vast majority of focus group participants could not remember ever seeing the existing physical activity and sedentary behaviour guidelines, and often were not aware of where to access them. As one stakeholder (Researcher/Parent;F;I:E) explained: I’ve been doing work trying to increase education around movement in child care centres. I’ve realized over the last 5 years or so that a lot of child care staff aren’t currently aware of our present physical activity guidelines for the early years. (page137). | Unequivocal |
| Brownson et al (48) | Low participation in dissemination activity. | Few respondents (2.1%) had participated in trainings specifically to learn about the Community Guide (QZ).(page s69). | Not Applicable – Qualitized finding |
| Mattran et al (47) | About 22% of participants have reported that they were not aware of the materials before taking the survey. | | Not Applicable – Qualitized finding |

1. *Facilitators*

The following table lists the findings extracted from each study related to the user-group, deemed as facilitators with its correspondent code.

| **Author** | **Codes** | **Findings with illustration** | **Quality of finding** |
| --- | --- | --- | --- |
| Mitton et al (49) | Identifying champions/leaders | Respondents felt that, from among this group of key players, it would be important to identify the leaders or champions who could help communicate research findings and facilitate KTE. These need not be persons in formal leadership positions. "When you actually look at what the common denominator is across a whole heterogeneous mix of KTE [pause] a successful KTE initiative, it is very often focused around a single person [pause] who had been charismatic, taken leadership, done championing, so on. So finding those people, identifying champions and leaders for this kind of activity and then being able to resource them, may in fact be the single most effective thing you could do in all of this" (P2). "You can say what you like in terms of knowledge but generally in terms of practice there are leaders and there are followers and if you get some leaders and champions on side, people's attitudes and behaviors may change" (P12). (page 3) | Unequivocal |
|  | Identifying champions/leaders | Having a lead person or key contact to manage the interface between researchers and decision makers was felt by some to be a key step. "you need a specific individual identified as your dissemination manager and that individual helps working with the researchers all the way through from the start to the end of the project" (P1). (page 5) | Unequivocal |
| Brown et al (58) | Accepting knowledge broker support | Last, they described the value of previous experience working in schools and/or school health research and that many schools were open to receiving support. "I think part of what has made this a useful endeavor is the appetite that schools seem to have, mostly, for this type of support. I think a lot of the schools really love having a knowledge broker to help them sort through stuff, or to meet with public health, or to help them with grant applications, things like that. So I think that has helped the process along". (Project manager). (page 596) |  |
|  | Valuing health | Researchers highlighted the importance of school staff, especially the school administrator, valuing student health. “I think where KB does really work, in a situation where there are really committed people on the school end, that really do want to make some sort of positive change within the school” (Knowledge Broker 1 [KB1]). “And almost always, it had to be an administrator. Because at times we’d get a phys. ed. teacher that was really excited, but it didn’t go anywhere because their administrator was kind of the gatekeeper to change” (KB2). (page 594) | Unequivocal |
|  | Valuing health | The principal assigning value to student health and a strong relationship between the school and public health personnel. "I feel that because I involve myself so much . . . and the staff see that this is valuable to me . . . the time that [teachers are] going to give [their] classes and allow [their] classes to do [the survey], that there is value to it. And that the results will be used in some way, it’s just not another task. (Principal, School 1 [S1])" (page 593) | Unequivocal |
| Evenson et al (45) | Participating in training | Participants primarily learned of the NPAP through communications from the NSPAPPH, the CDC, and at national and state meetings. Several interviewees also helped with the plan creation, while others learned of it when hired into their position. Most respondents shared the NPAP with others, often involving further discussion such as through webinars or meetings. For example, one interviewee “did a state wide seminar when it first came out” and another stated that “the first thing I did after the plan was rolled out was I brought together … seven different departments here at the state … and I educated them on the plan and just basically did a state rollout here. And then we also sent this similar information and held a teleconference call for the counties.” (page 3) | Unequivocal |
| Dagenais et al (57) | Valuing health and research  Organisation valuing health | The projects were important to the IPPs because the projects’ mandates fell within their organizations’ priorities, or because the partners’ stakeholders (workers or patients) wanted the research to be done, or because their stakeholders valued evidence-based practice and investment in research. Some individual interviewees stated that their project’s focus went beyond their organizational mandate and was actually a personal passion. “This particular project, it was one that we were absolutely proud to be a part of and felt that it was just giving us more capacity. In fact, it was fulfilling our mandate in a very thoughtful way with very skilled operators. It allowed us to hit areas we would have liked to be hitting but just hadn’t gotten to.” (pg 272) | Unequivocal |
| Riazi et al (46) | Playing an influential role | Like physicians, ECEs recognized the important role they played in sharing health and wellness information, and were keen to play their part. Female ECE (Early Childhood Educator;F;FG;E): "Preschool teachers…have so much clout and so much… Interviewer: They hold influence… Female ECE: …influence. I never actually realized it until…I’m only, I’m new as a Strong Start facilitator, so only a couple of years, and it’s just amazing how much parents really look to you." (page 140). | Unequivocal |
| Brownson et al (48) | Valuing research | The majority of the administrators and managers (n=40, 81.6%) were supportive of evidence-based interventions. (QZ). (page s69) | Not applicable - Qualitized finding |
|  | Valuing health and research | Regarding the organizational climate, the majority of respondents reported that their health department was supportive of evidence-based physical activity promotion (n=32, 65.3%). (QZ) (page s69) | Not applicable - Qualitized finding |
|  | Prior awareness of guide. | Awareness of the Community Guide among administrators and managers was relatively high (67.3%); (page s69)(46) (QZ) | Not applicable - Qualitized finding |
|  | Prior awareness of guide. | Awareness of the Community Guide among respondents was also high (89.8%).(page s69) (QZ) | Not applicable - Qualitized finding |
|  | Prior awareness of guide through website. | Most survey participants (67.3%) had visited the Community Guide website. (page s69) (QZ) | Not applicable - Qualitized finding |
|  | Interest in participating in training | Survey respondents showed a strong interest in training sessions for increasing the use of evidence-based strategies to promote physical activity (data not shown). Nearly all participants indicated that they would take part in such training (95.9%) or would send staff to training (97.9%). Pg S69 (QZ) | Not applicable - Qualitized finding |
|  | Onsite workshop as preferred mode of dissemination | Respondents were also asked about the preferred mode of training or technical assistance. Using a 1–5 rating scale, the most popular option was an onsite workshop (mean score=4.1) (page s69) (QZ) | Not applicable - Qualitized finding |
|  | Telephone help line as second preferred mode of dissemination | The second preferred mode of training or technical assistance was using a telephone help line (mean score=3.5). (page s69) (QZ) | Not applicable - Qualitized finding |
|  | Expert to answer questions as third preferred mode of dissemination | The third preferred mode of training or technical assistance was an expert being available to answer questions (mean score=4.0) (page s69). (QZ) | Not applicable - Qualitized finding |
|  | Grant writing as fourth preferred mode of dissemination | The fourth preferred mode of training or technical assistance was help with grant writing (mean score=3.9). (page s69). (QZ) | Not applicable - Qualitized finding |
|  | Using a CD-ROM as fifth preferred mode of training | The fifth preferred mode of training or technical assistance was help using a CD-ROM (mean score=3.7). (page s69). (QZ) | Not applicable - Qualitized finding |

1. **The issue**
   1. *Barriers*

The following table lists the findings extracted from each study related to the issue, deemed as barriers with its correspondent code.

| Author | Codes | Findings with illustration | Quality of finding |
| --- | --- | --- | --- |
| Mitton et al (49) | Hard to link research back to policy | Several respondents suggested that there were differences between KTE involving research-to-clinical practice, and that involving research-to-policy, with the latter being a more problematic or less understood opportunity. "Less is linked back to policy.... I think [information] gets lost and stays at the clinical or at the scientific level. And they do a good job, moving that information around at that tier. It has a very hard time coming through the glass ceiling though, into the policy world" (Participant 7). (page 5) | Unequivocal |
| Brown et al (58) | Perceived difficulty of researchers to communicate with uninterested end-users. | Researchers found it difficult when schools were unresponsive to their requests or were not interested in participating (n=4)(p597). “If [schools] don’t want our help, then we obviously have a challenge in communicating with them, keeping the dialogue going” (KB5)” | Unequivocal |
|  | Researchers’ lack of clarity in setting tasks and roles for knowledge brokers | Researchers discussed the challenge of defining tasks and expectations for the knowledge broker role (p597). "There’s also a limit to what I can do as well, with my own studies and own limitations of schools being far away, so how much time can I realistically put into driving there and helping to coordinate things when I’m one person too? . . . And how for it should extend? (KB6)" |  |

- 1. *Facilitators*

The following table lists the findings extracted from each study related to the issue, deemed as facilitators with its correspondent code.

| Author | Codes | Findings with illustration | Quality of finding |
| --- | --- | --- | --- |
| Brownson et al (48) | Promoting physical activity is high priority for health department | Organizational climate - Promoting physical activity is high priority for health department n=32, 65.3%. Pg s69. (QZ) | Not applicable - Qualitized finding |

1. **The research**
   1. *Barriers*

The following table lists the findings extracted from each study related to the research, deemed as barriers with its correspondent code.

| Author | Codes | Findings with illustration | Quality of finding |
| --- | --- | --- | --- |
| Faulkner et al (44) | Content of guidelines does not include behaviour of interest. | Receptivity: Fitting things together like one big puzzle - Multiple stakeholders also commented on the disconnect of the current guidelines and how movement is not strictly based on the 2 ends of the continuum (i.e., very active vs. very sedentary). "Sometimes we talk a bit more to physical activity, we talk about sedentary behaviour, we don’t necessarily talk about that light physical activity … I think having that idea of integrating all of those things into our day is a positive thing. because we focus so much on the end of the spectrum and not necessarily on that other part that fills a large chunk of our day. (EXP;F;FG;E)".Pg S305. | Unequivocal |
|  | End-users’ lack of understanding about content of guidelines. | The most prominent barrier to the Movement Guidelines was potential confusion with understanding terms such as MVPA [moderate-to-vigorous physical activity], LPA [light physical activity], and recreational screen time - The majority of participants recognized that they themselves and children and youth may not fully understand the meaning of MVPA.  "If you ask them [students] to try and identify it [MVPA] and use it in their own language it’s hard for them to explain … There are some [teachers] that don’t necessarily understand it [MVPA] themselves. There are maybe opportunities where they [teachers] think that they’re getting their kids moving within a qualification of moderate to vigorous activity but really it isn’t. (TEA;M;I;E).". Pg S306.  “perception of what moderate to intense is, or intense workouts and activity, may not [be] well understood by the general public” (EXP;F;FG;E). Pg S306. Many participants were not aware that the suggestion of “1 hour of MVPA” could be accumulated throughout the day rather than completed all at once. PgS306. | Unequivocal |
|  | *End-users* are confused with technical physical activity terms and proportion of recommended physical activity levels of guidelines. | Potential confusion with understanding terms such as MVPA, LPA, and recreational screen time-some participants suggested that an integrated guideline, which includes behaviours that are not clear. This was specifically addressed towards the portion of MVPA given that it is such a small percentage of a 24-hour day has “the potential to lose some of the key points” (PED;M;I;E). “The one thing that’s striking that may actually be a good thing for some people and maybe less of a help for others is that the targeted area of the moderate to vigorous physical activity is such a small slice of the pie. On the one hand for somebody who’s not very active to look at it and go “oh I’m not doing so badly”. But it looks like it is such a tiny proportion that the importance of it appears to be diminished because it doesn’t represent a big part of the guideline. (PED; F;FG;E). Pg S306. | Unequivocal |
| Ritchie et al (53) | Perceived important risk factors by end-users are missing from the guide. | Out of 28 participants, five have suggested that the omission of certain environmental risk factors hinder the ECAC’s promotion. Pg 4. (QZ) | Not applicable - Qualitized finding |
|  | Giving the same importance to all messages included in guide. | Out of 28 participants, four suggested that the presentation of ECAC suggests all 12 messages are equivalent in their importance to the cancer burden and it hinders its promotion. Pg 4.(QZ). | Not applicable - Qualitized finding |
| Evenson et al (45) | Lack of compatibility between plan and government goals and recommendations | Not compatible or consistent: some respondents noted the NPAP was compatible with their existing state plan or goals, but others said it lacked compatibility with federal policy recommendations. “There seems to be a disconnect with maybe some of the federal policy recommendations and the Physical Activity Plan…. I think that the other federal organizations could do a better job of supporting that at the national level.”. Pg 4. (32) | Unequivocal |

- 1. Facilitators

The following table lists the findings extracted from each study related to the research, deemed as facilitators with its correspondent code.

| Author | Codes | Findings with illustration | Quality of finding |
| --- | --- | --- | --- |
| Faulkner et al (44) | Including comprehensive content | Understand the interplay of the movement behaviours and further achieve healthy lifestyle habits - Similar to other focus group participants, a paediatrician discussed the benefits of including information related to sleep: "I think it’s important to balance both sides of the equation … If you don’t sleep, well you don’t have as much energy to expend or to conduct the activities of your day. I think you’re less likely to engage in physical activity. We know that there are links between sedentary behaviour, sleep, and obesity risk as well. So a lot of the outcomes that you’re targeting with this [guideline] are going to be affected by sleep as well. So it all kind of fits together as one big puzzle. (PED;F;FG;E).Facilitator. Pg S305. | Unequivocal |
| Ritchie et al (53) | Including comprehensive content | Out of 28 participants, fifteen cited the comprehensiveness of the cancer prevention topics addressed as a positive internal factor. | Not applicable - Qualitized finding |
|  | Including robust evidence | Out of 28 participants, eleven cited the robust evidence base underpinning the recommendations as a positive internal factor. | Not applicable - Qualitized finding |
|  | Highlighting important content.  Consistently including content. | Out of 28 participants, five cited the prominence given to the main modifiable risk factors as a positive internal factor which four participants have noted that they were emphasised consistently in each edition of the ECAC. | Not applicable - Qualitized finding |
| Riazi et al (46) | Clarity, conciseness, and systematic presentation of content | Across all interviews and focus groups, participants applauded the clarity and conciseness of the guidelines. As one stakeholder (Researcher;F;I;E) explained, “I thought they were fairly clear, concise, and systematic in their presentation”. FACILITATOR (p136). | Unequivocal |
|  | Presentation of information easier to access | Participants liked that the information was divided based on age (i.e., infants, toddlers, and preschoolers), making it easier to access. One participant (Physical Activity Communicator;F;I;E) pointed out that “breaking it up into the 3 categories makes it more usable for people working in this area”. (p136) | Unequivocal |
|  | Including relevant content | Discussion around the utility of the guidelines revealed that well-received components of the Movement Guidelines included the provision of specific and concrete goals, the inclusion of sleep and quality sedentary activities (e.g., reading, drawing), and the recommendation for replacing indoor with outdoor time. "I think the actual existence of it when it’s completed would be really helpful for me because often, parents ask over the years, “How long should my child be sleeping?” or “What do you think?” and we give educated guesses. To me, it’s helpful to have something to refer to or to send parents to.” (P137) | Unequivocal |

1. **The researcher-user relationship**
   1. Barriers

The following table lists the findings extracted from each study related to the researcher-user relationship, deemed as barriers with its correspondent code.

| Author | Codes | Findings with illustration | Quality of finding |
| --- | --- | --- | --- |
| Haynes et al (52) | Lacking common priorities | A significant enabler that emerged was the need for mutual respect for the different ways that practitioners go about their day-to-day business in comparison to researchers. This led to some difficulties with alignment of the partners and the researchers, especially at the beginning of the partnerships. For example: “It is more like oil and water. It can exist in the same bucket but you never fully integrate. “There was definitely a bit of a bump, bumping along you know, when you realized that you don’t share a lot of the same vocabulary and you don’t share a lot of the same working priorities and all those kind of things”. Page 271 | Unequivocal |
|  | Partnership lacking leadership | Right partners involved- There were a few instances where interviewees felt that the wrong person from the organization had been included in the project, and that this had affected the organization’s ability to use the knowledge effectively. “One of the keys to success of these types of partnerships is everyone’s mandate and who has the authority to affect change in that mandate or give it credence, right? So you did this work but what was the uptake? What was the outcome?”. Page 269 | Credible |
|  | Organisation’s priorities weight more over potential members of partnership | In the two projects that did not include government decision-makers as partners, the value of including policy decision-makers as part of the research team was not clear. Some thought it would be better to only involve government as an IPP once results were finalized. Another thought that it would be difficult to engage a policy maker sufficiently without overwhelming them with work. “I would say [policy makers] were probably missing but were they needed? ... They can say so much ... but the broader priorities of the organization take precedence over what they might say ... . Also, how do we want to present it to government? ... We can be engaging with bureaucrats but we can also engage with the elected officials and sometimes there are other means of getting policy changes.”. Page 269-270 | Unequivocal |
| Brown et al (58) | Lacking awareness of existent partnerships. | Importance of strong relationship between public health unit and school "I don’t know if all schools that are involved with COMPASS connect with public health. . . . Or if there’s as close a relationship between public health nurses and administrators at schools as we have in [community name]. (Public health nurse)". Page 593 | Credible |

- 1. Facilitators

The following table lists the findings extracted from each study related to the researcher-user relationship, deemed as facilitators with its correspondent code.

| Author | Codes | Findings with illustration | Quality of finding |
| --- | --- | --- | --- |
| Haynes et al (52) | Working with an established relationship | Many of the partners had worked with the researchers in the past. In a few cases, the pre-existing relationship helped the organizations make the decision to sign on as a partner.  “Going into [the project] we pretty much had established the relationship in my view. I was very familiar with your work, with the type of people that you were, your passion for it ... . Of course we’re going to at every opportunity partner with you guys.” Page 270. | Unequivocal |
|  | Having an opened and respectful interaction between partners | Partners spoke about the project leadership’s openness to everyone’s ideas and input. “[The project lead] is very open-minded, very friendly, very easy to engage with. S/he’s responsive and respectful. That’s important in providing the kind of leadership s/he has to provide. At the same time, s/he’s not a doormat ... . S/he has [their] beliefs in how things need to move forward and has the evidence to back it up. So you respect what s/he’s coming forward with. And if s/he doesn’t agree with a position s/he’s comfortable saying that and why. So it’s been a nice balance.” Page 270. | Unequivocal |
|  | Having credible and independent project leaders | Partners also thought that the project leaders were credible, based on their previous research experience or the organizations from which they came. “[They’re] not there working for industry. [They’re] independent, really. And where do you find that? You don’t ... . You can’t find that anywhere. So yeah, we would latch on ... . I’m a big fan.” Page 270 | Unequivocal |
|  | Mutual respect and communication key for a successful partnership | Many partners acknowledged that mutual respect is important for a successful partnership. “I would say some of the keys to a good partnership are to be able to communicate and to be able to understand each other’s point of view and respecting each person’s unique differences but also knowing when to leverage their strengths.” Page 270 | Unequivocal |
|  | Respect and gratitude for the knowledge and skills from each partner  Parties benefiting from partnership | Mutual respect- Respect for the other’s knowledge and expertise extended to gratitude for being able to benefit from the specialized knowledge and skill that each partner brought to the table. Partners also knew their own specialized skill set and what they were able to bring to the table. “When I met [the project lead]... he gave us an overview of the project, and I expressed interest. Could we tap into it? Both from the angle of being involved in the project and contributing to it but also being able to utilize the resources? ... The work being done by the group, that’s been a great boon to us because they did all that review stuff that we normally do on our side. It just allows us to be lazy basically! In a really good way ... . The stuff that’s been developed is great stuff. We can tap into that and utilize that.” Page 271. | Unequivocal |
|  | Strategic engagement with decision-makers | In the two projects that did not include government decision-makers as partners, the value of including policy decision-makers as part of the research team was not clear. Some thought it would be better to only involve government as an IPP once results were finalized. Another thought that it would be difficult to engage a policy maker sufficiently without overwhelming them with work. I would say [policymakers] were probably missing but were they needed? ... They can say so much ... but the broader priorities of the organization take precedence over what they might say ... . Also, how do we want to present it to government? ... We can be engaging with bureaucrats but we can also engage with the elected officials and sometimes there are other means of getting policy changes.”. Page 269-270. | Unequivocal |
| Mitton et al (49) | Having frequent interaction.  Existing long-term relationship. | Informants argued that effective KTE is built upon longterm, personal relationships between decision makers and researchers. On-going interaction allows both groups to gain better understanding of each others' worlds, and the pattern of incentives and constraints which each face. " [What] is necessary is to have a continual interactive dialogue between the policy maker and the researcher about the question that is being raised and being researched, because as the researcher gets into understanding what some of the background is to the particular topic, that brings up new kinds of information that then informs the policy maker" (Participant 1). "... you have to have some decision maker, some policy maker, some users of health information attached to your group from day one, so they can (a) help you frame the research questions, (b) tell you how these research questions fit into the policy or decision-making environment and why they are important there, and (c) when you've done your research and when the knowledge is ready to sort of disseminate or transfer, they already have a receptor for that dissemination to happen or for that transfer to happen." (Participant 6) - (Page 3) | Unequivocal |
|  | Having close engagement, trust and joint decision-making.  Conducting participatory research. | The close engagement and joint decision making advocated in KTE are also principles of participatory research methods. Researchers who work in this vein may find it easier to achieve the relationships conducive to effective KTE. "More of the work I have done in the last five years has been involved in participatory research where the researcher knows us, knows the Centre, still has some of the distance to be able to do some of their work, but I think some of the distance in traditional research is artificial, and I think it gets in the way of some of the knowledge transfer. So I think the fact that I have colleagues that I work with and trust, and know me... has made a huge difference." (Participant 15) - (Page 3) | Unequivocal |
|  | Creating networks to reach stakeholders. | Because of limited networking opportunities, many participants tended to draw on the information they had immediate access to rather than approaching other organizations. "Certain challenges exist within different departments in the same organization and other organizations like they expect us to fill the form describing the information needed and [we] expect to hear back after a number of days. [Now] I am thankful with the networking that TROPIC started as we meet and [know] the people that we [usually struggled] to see within the Ministry and those outside the Ministry and is not a challenge any more." | Unequivocal |

1. Dissemination strategies
   1. Barriers

The following table lists the findings extracted from each study related to the dissemination strategies, deemed as barriers with its correspondent code.

| Author | Codes | Findings with illustration | Quality of finding |
| --- | --- | --- | --- |
| Faulkner et al (44) | Specialists cannot reach a broad audience | Doctors, paediatricians, and nurses were also discussed as important messengers for communicating the Movement Guidelines. These health care practitioners were regarded as trustworthy and respected sources; however, they may not have the time to interact and explain the Movement Guidelines to all families. One paediatrician explained the main challenge of her practice: "We’re not going to be seeing – at least I think – the healthy kids. We’re not going to be targeting the “healthy” children … a community paediatrician might have a very different take on it. But somebody who works exclusively in a hospital is going to see a very small subset." (PED;F;FG;E). (p308). | Unequivocal |
| Ritchie et al (53) | Addressing the general population with the “one-size-fits-all” approach | Uniformity of audience: When considering internal factors hindering its promotion, promoters most frequently noted the “one-size-fits-all” approach towards addressing the general population (n = 13)(pg4). | Not applicable - Qualitized finding |
|  | Standardising messages in plan | Uniformity of messages: the standardisation of the 12 messages of ECAC was identified as a hindrance (n = 10) (pg4). | Not applicable - Qualitized finding |
|  | Plan disseminated is too lengthy.  Omitting content perceived as important. | Disadvantages of the ECAC influencing its promotion and dissemination: Several promoters […] felt that the length of the 4th edition (n = 7) and omission of certain environmental risk factors (n = 5) created difficulties in the promotion of the ECAC(pg4). | Not applicable - Qualitized finding |
| Brown et al (58) | Undervaluing knowledge brokering outputs | The most frequently mentioned challenge associated with knowledge brokering was record keeping. Initially, researchers did not realize the value of information generated from knowledge brokering and hence did not implement systematic record-keeping procedures. "We were coming up with this more or less as we went along. And it becomes an afterthought sometimes, to say, “we’ve got all these notes but how are we storing them, how are we presenting them to people, how are we making them user friendly?” And the answer was we weren’t doing a very good job of that. (Project manager)". | Unequivocal |
|  | Lacking consistent knowledge brokering procedures. | Record keeping. Knowledge brokers unsure as to whether they are recording notes the same way as others (n=3). “I think we could probably be charting our calls a little better. . . . I think my notes are pretty good, but that’s just how I’ve been doing it” (KB1). (p597). | Unequivocal |
| Evenson et al (45) | Inability to identify the targeted audience. | Complexity: some respondents described the NPAP as being concise, while others said it provided a broad overview but did not help with implementation, making it more difficult to use. “I still wonder how we all want to see this really being effective and who is it for? And I think those are huge questions, like who is this really for? If it’s for the general public, then we really need to nail it down. If it’s really kind of a document for people like you and I that do this work, then I think the format that it is, is going to work fine.” Pg 4. | Unequivocal |
| Haynes et al (52) | Uncertain dissemination timing | Communication - The IPPs on the Burden of Occupational Cancer project did appreciate the efforts that the principal investigators (PIs) made to maintain strong communication (they scheduled regular working breakfasts and lunches together). There was some confusion regarding who was supposed to be leading the planning of conferences. Also, the IPPs wished they had received more regular updates on the process and a clearer understanding of when the research findings could be shared publicly. They were anxious to use the burden estimates for occupational asbestos exposure in their advocacy work.  “There was a time early on [when we said],‘When can we use these [asbestos] numbers because we would like to start incorporating them?’ and the PI was like, ‘Well they’re still not final ... you have to wait.’ From a research perspective, recognize that you need to have these numbers complete and validated. [They were] more like, ‘We’re showing you these [other] numbers. Here’s the diesel. Here’s the wood dust. You can start using these in your work.’ We didn’t have the appetite or the resource really to take on those new areas.”. Pg 271. | Unequivocal |
| Williams et al (51) | Information was too technical, short and not easy to read | Participants most commonly reported that the section of the packet on the Motivational Interviewing Evidence Base and Effectiveness is what they liked the least about the motivational interviewing packet. Specifically, participants reported that the information on CER was too technical and contained too many acronyms and too much data, making it difficult for the reader to understand. For example, one participant reported, ‘I got a little lost with the tables explaining the characteristics of CER studies of motivational interviewing. A paragraph would have been satisfactory’. Another participant reported, ‘Characteristics of the CER studies (the tables), I’d rather just a summary of what the studies found’. The participants felt the inclusion of research evidence is important, but should be used sparingly. p91. | Unequivocal |

- 1. Facilitators

The following table lists the findings extracted from each study related to the dissemination strategies, deemed as facilitators with its correspondent code

| Author | Codes | Findings with illustration | Quality of finding |
| --- | --- | --- | --- |
| Brown et al (58) | Presence of knowledge broker | Being involved with other aspects of the study (i.e., data collection, recruitment)(N=4). “I’ve gone to some data collections of schools that I knowledge broker for, so I couldn’t really do a KB meeting on the spot because we didn’t have the data, but I got to know my contact there” (KB5). | Unequivocal |
|  | Knowledge brokers working as a team | Facilitators focused on internal components of the study such as communicating with other knowledge brokers. “The knowledge brokers each have their schools that they are responsible for, but they also work as a team. So they do bounce ideas off each other as well—that, I think, is helpful” (Project manager). | Unequivocal |
|  | Approachable knowledge brokers | When discussing facilitators to knowledge brokering participation, knowledge users and researchers focused on different aspects. Knowledge users described characteristics of knowledge brokers (i.e., approachability, availability, and expertise): She was very approachable in the sense that I could feel like I could ask her anything, I was comfortable when speaking with her, and she offered a lot of other ideas as well, like when she would get in touch with me or vice versa, she was always able to offer suggestions. (Teacher, S5). | Unequivocal |
| Ritchie et al (53) | Endorsement and coordination by recognised organisations provides credibility | The coordination and endorsement of the International Agency for Research on Cancer (IARC) (n = 5) and the European Commission (n = 5) provides important credibility. | Not applicable - Qualitized finding |
| Mitton et al (49) | Building communities of practice | Thus, they recommended strategies which would foster and grow social networks and communities of practice around new evidence and best practices identified through the ADI [Alberta Depression Initiative]. "Well, first of all you have got to bring together the community of interest..... Number two, you have to identify whether they have a reason to come together and try and advance the practice of health interventions in dealing with depression. Third, bring them together as a community of practice.... Then I think you have got to bring them to the point where they are going to be functioning as a bit of a network..." (Participant 1). (Page 4) | Unequivocal |
|  | Developing plans to transfer information with key stakeholders | The aim of the proposed KTE strategy for the ADI [Alberta Depression Initiative] is to enable transfer and exchange of information among key stakeholders in order to positively impact depression research, practice, and policy making. In the opinion of our interviewees, effective approaches to KTE need to be carefully thought out and planned in advance. “Think the whole chain out – what do you really want to achieve with your message to a policy maker? You must think the whole chain through and not only be clever in putting it, making a summary on one page, or to send it in terms of guidelines, you have to think all the way up to what you want to achieve at the end. And think those steps out and take action on all of them" (Participant 13). "I think first of all it needs to be something that is clearly developed. It has to have who it is aimed at, who it is targeted at, what are the goals and the objectives" (Participant 12). (Page 6) | Unequivocal |
|  | Interacting face-to-face. | The centre of the proposed approach is a facilitated workshop of key mental health stakeholders in Alberta, with specific pre- and post-workshop activity. The workshop model was specifically endorsed by several respondents. One interviewee provided a succinct summary of the benefits that such a model would have, in light of the suggested KTE strategies described above: "I think we are seeing [workshops] more and more and I think that can be very effective. It can be a good use of a fairly small amount of time although if you count up all the hours of the participants it is not insignificant, but you get everyone on the same page pretty quickly, let them know what is happening and then let them work for 4 to 6 hours and you can get a fair bit done. So I think we are tending to want to use that format. I think the other thing it does is put people into face-to-face contact and you can't get the same kind of interaction through other means and I just think the buy in to decisions is much more, the understanding of the complexity of each other's world is much more ... you start to understand the realities of everyone's world [and] then I think that you get some very creative solutions" (Participant 3). (Page 6) | Unequivocal |
|  | Including key stakeholders in the network | Also in their view, these networks should include not just researchers and health sector organizations like health authorities, but the larger community sector as well. A community agency director, with decades of experience working in a health authority and a university, spoke of the neglect of the community in KTE efforts, despite the knowledge, experience, capacity and interest. "I think one of the critical pieces here is the exchange strategy including the community, because so often what happens is it only includes the institutions. By that I mean the formal mental health system, the Health Region, and the University... and those of us in the community that are doing the bulk of the work are left out of this. And it is not back and forth. It is usually... 'we, the institution, know and it is you folks in the community that are the recipients'... and sometimes [community agencies] have a lot to offer that the institutions don't." (Participant 15). (Page5) | Unequivocal |
| Evenson et al (45) | Continuing diffusion of information | “We’ve put it out in our email listserv to local public health and others interested in physical activity." Page 3. | Unequivocal |
|  | Continuing diffusion of information | Awareness of the NPAP: Most respondents shared the NPAP with others, often involving further discussion such as through webinars or meeting. For example, one interviewee “did a state wide seminar when it first came out” and another stated that “the first thing I did after the plan was rolled out was I brought together … seven different departments here at the state … and I educated them on the plan and just basically did a state rollout here. And then we also sent this similar information and held a teleconference call for the counties.” Page 3. | Unequivocal |
| Haynes et al (52) | Relatable story-telling. | Mutual respect- The partners knew, from their experience in policy advocacy, that they needed to strategize and find the best way to communicate the numbers. “Research really helps inform what our policy position would be. But even when you have the research, you still have the personal story. You know, the real-life example of it. It’s really important to help when you’re talking to policymakers, especially... Like when you can bring it to life by having a real story to say, ‘This is why this is really important to make this policy change.” Page 271. | Unequivocal |
| Waqa et al (54) | Supportive and present knowledge brokers.  Interacting face-to-face. | The various forms of motivating strategies employed by the TROPIC knowledge-brokering team included both face-to-face group workshops and group meetings, both of which focused on critical appraisal of other policy briefs. However, the one to one meetings were the preferred method for most participants. Many participants commented on the reliability of the TROPIC team. "[The] motivating strategies have been good ……… when we are stuck in our policy writing….., they always come in and assist… and make sure that we keep on moving…" | Unequivocal |
|  | Creating networks to increase connections between stakeholders | Because of limited networking opportunities, many participants tended to draw on the information they had immediate access to rather than approaching other organizations. "Certain challenges exist within different departments in the same organization and other organizations like they expect us to fill the form describing the information needed and [we] expect to hear back after a number of days. [Now] I am thankful with the networking that TROPIC started as we meet and [know] the people that we [usually struggled] to see within the Ministry and those outside the Ministry and is not a challenge any more." |  |
| Riazi et al (46) | Including a wholistic approach to present information | Similar to other participants, a researcher agreed that a 24-h perspective for the Movement Guidelines was useful" My first impression was that it was a good idea to go to the 24-hour movement approach. It makes a lot of sense from a public health messaging perspective to deliver the messaging in the context of what a healthy day looks like. This is an advantage of the current guidelines." (Researcher;M;I;E) (Page 137). | Unequivocal |
|  | Choosing appropriate settings to disseminate information | Child care settings were seen as another natural conduit for sharing the Movement Guidelines as these settings serve families with infants, toddlers, and preschoolers. ECEs and daycare staff generally said they endeavoured to build trusting relationships with families, often shared information about activities and initiatives that could be reinforced at home (e.g., literacy techniques), and considered themselves as having a “huge role in child development” (Early Childhood Educator;F;FG;E). One ECE (Early Childhood Educator;F;FG;E) said: I feel like our role is mostly liaising with the parents. Maybe trying to notice where there might be gaps and knowledge of what’s needed at that age and volunteering information when it might be useful. (Page 140) | Unequivocal |
|  | Disseminating information through existing and appropriate structures | With many settings and programs already devoted to the early years, there was consensus that the dissemination and, to some extent, the implementation of the Movement Guidelines should be integrated within existing communication channels. As another example, pre- and post-natal classes at hospitals and public health units could introduce new parents to the Movement Guidelines in their sessions: “The guidelines could be embedded within prenatal care, to motivate people to start thinking about how to structure the day before even having children” (Researcher;M;I;E). (Page 140) | Unequivocal |
|  | Disseminating information through existing and appropriate structures | Information about the Movement Guidelines could be made... “…available to nurses who are going into homes of moms with babies and helping them with sleep, breastfeeding, or formula feeding. The guidelines can also be part of the information that is going to moms, and nurses may [even] model the appropriate behaviours” (Physician;F;I;E). (Page 141) | Unequivocal |
|  | Disseminating information through existing and appropriate structures | A physical activity advocate also suggested that existing workshops for parents could be updated to include the recommendations. "Public health units are amazing and they generally have a physical activity promoter on staff, if not more than one, and also a child and family health manager. They have a mandate to do workshops and to connect with parents. Those would be good avenues [for dissemination]. (Physical Activity Communicator;F;I;E) (Page 141) | Unequivocal |
|  | Disseminating information through existing and appropriate structures | Participants working in physical activity promotion also used electronic media to promote programs and initiatives. One participant (Physical Activity Communicator;- F;I;E) explained, “In the webinars that we do, we could leverage the new guidelines, and definitely through our communications we can start some conversations around these guidelines”. Page 141 | Unequivocal |
|  | Disseminating information through existing and appropriate structures | One final suggestion for disseminating the Movement Guidelines in an electronic format was an online training module for ECEs and primary school teachers. A researcher-informant (Researcher;F;I;E) noted, “The most fiscally responsible time and logical time [to disseminate information about the guidelines] is [physical activity] training within the ECE college curriculum before they enter the field” and that an “online module that ECE programs can pick up and administer to their students prior to leaving the college” was an ideal way of equipping educators with the skills to facilitate energetic play in daycare settings. Such a module could also be useful for ongoing professional development of teachers as well as license renewal for ECEs. Page 141 | Unequivocal |
|  | Adapting resources to audience | One suggestion from the key stakeholder interviews was the development of ready-to-use-tools such as workshop outlines with practical strategies, discussion points, and activities geared toward ECEs. As explained by a physical activity educator (Physical Activity Communicator;F;I;E), “If there were resources that showed what the guidelines look like in practice in homes, daycares, at a community centre, in a community in general, that would be helpful”. (Page 141) | Unequivocal |
| Mattran et al (47) | PDF files helping raise awareness | About 38% of participants have reported that they were aware of the materials through links to PDF files from the NSPAPPH [National Society of Physical Activity Practitioners in Public Health] Website before receiving the survey. | Not applicable - Qualitized finding |
|  | Email subscription helping raise awareness | About 48% of participants have reported that they were aware of the materials through e-mail subscription/list-serve before receiving the survey. | Not applicable - Qualitized finding |
|  | Link to PDF files helping to raise awareness | About 22% of participants have reported that they were aware of the materials through links to PDF files from the CDC [Centers for Disease Control] DNPAO [Division of Nutrition, Physical Activity and Obesity] Website before receiving the survey. | Not applicable - Qualitized finding |
|  | Institution contacts helping raise awareness | About 13% of participants have reported that they were aware of the materials through CDC contacts before receiving the survey. | Not applicable - Qualitized finding |
|  | Government contacts helped raise awareness among very few participants | About 5% of participants have reported that they were aware of the materials through State or local health department contacts before receiving the survey. | Not applicable - Qualitized finding |
|  | Agencies helped raise awareness among very few participants | About 4% of participants have reported that they were aware of the materials through Federal agency before receiving the survey. | Not applicable - Qualitized finding |
|  | Non-profit organisation contacts helped raise awareness among very few participants | About 4% of participants have reported that they were aware of the materials through nonprofit organization contacts before receiving the survey. | Not applicable - Qualitized finding |
|  | Word of mouth/news story helped raise awareness among very few participants | About 4% of participants have reported that they were aware of the materials through word of mouth or media/news story before receiving the survey. | Not applicable - Qualitized finding |
|  | Search engine helped raise awareness among very few participants | About 4% of participants have reported that they were aware of the materials through search engine before receiving the survey. | Not applicable - Qualitized finding |
|  | Website helped raise awareness among very few participants | About 2% of participants have reported that they were aware of the materials through referral or link from a non-CDC and non-NSPAPPH Website before receiving the survey. | Not applicable - Qualitized finding |
|  | Doctor or allied health professional helped raise awareness among very few participants | About 3 participants have reported that they were aware of the materials through information from doctor or allied health professional before receiving the survey. | Not applicable - Qualitized finding |
| Williams et al (51) | Information disseminated was about right. | About 85% of participants reported that the content on the Motivational Interviewing [MI] packet was about right | Not applicable - Qualitized finding |
|  | Adapting content to audience | About 49% of participants reported that the Motivational Interviewing [MI] packet was somewhat user-friendly. | Not applicable - Qualitized finding |
| McBride et al (55) | Workshops as the preferred dissemination channel | Evaluation of teacher educator workshops revealed that 88.8% of teacher educators strongly agreed or agreed that the workshop was a good way of equipping trainers with the necessary knowledge and skills to be able to train teachers to use the SHAHRP lessons as intended. | Not applicable - Qualitized finding |
