# Supplementary material for: Barriers and facilitators to dissemination of non-communicable diseases research: a mixed studies systematic review
Source: Front Public Health. 2024 Oct 2;12:1344907. doi: 10.3389/fpubh.2024.1344907 (PMC11479996; doi:10.3389/fpubh.2024.1344907)
Supplement: Supplementary file 5 [file Data_Sheet_5.docx]

Additional File 5

Data transformation process suggested by JBI

Qualitative data

Independently got familiarised with the data

Independently transformed the quantitative data into qualitized data

Met in pairs to discuss qualitization

Consolidated data to prepare for synthesis

Qualitized data (QZ)

Synthesis

Transformation
