# Supplementary material for: Barriers and facilitators to dissemination of non-communicable diseases research: a mixed studies systematic review
Source: Front Public Health. 2024 Oct 2;12:1344907. doi: 10.3389/fpubh.2024.1344907 (PMC11479996; doi:10.3389/fpubh.2024.1344907)
Supplement: Supplementary file 6 [file Data_Sheet_6.docx]

Additional File 6

Quantitative findings transformed into qualitized findings (QZ)

1. The user-group

| Verbatim quantitative findings | Qualitized findings |
| --- | --- |
| Administrators and managers supportive of evidence-based interventions n=40, 81.6%. (page s68-69)(48) | The majority of the administrators and managers (n=40, 81.6%) were supportive of evidence-based interventions. (QZ). |
| Regarding the organizational climate, the majority of respondents reported that their health department was supportive of evidence-based physical activity promotion (n=32, 65.3%). (page s69)(48) | Regarding the organizational climate, the majority of respondents reported that their health department was supportive of evidence-based physical activity promotion (n=32, 65.3%). (QZ) |
| Awareness of the Community Guide among administrators and managers was relatively high (67.3%) (page s69) (48) | Awareness of the Community Guide among administrators and managers was relatively high (67.3%).(QZ) |
| Awareness of the Community Guide among respondents was also high (89.8%).(page s69) (48) | Awareness of the Community Guide among respondents was also high (89.8%).(QZ) |
| Most survey participants (67.3%) had visited the Community Guide website. (page s69) (48) | Most survey participants (67.3%) had visited the Community Guide website. (QZ) |
| Few respondents (2.1%) had participated in trainings specifically to learn about the Community Guide. (page s69) (48) | Few respondents (2.1%) had participated in trainings specifically to learn about the Community Guide.(QZ) |
| Survey respondents showed a strong interest in training sessions for increasing the use of evidence-based strategies to promote physical activity (data not shown). Nearly all participants indicated that they would take part in such training (95.9%) or would send staff to training (97.9%). (page s69) (48) | Survey respondents showed a strong interest in training sessions for increasing the use of evidence-based strategies to promote physical activity (data not shown). Nearly all participants indicated that they would take part in such training (95.9%) or would send staff to training (97.9%).(QZ) |
| Respondents were also asked about the preferred mode of training or technical assistance. Using a 1–5 rating scale, the most popular option was an onsite workshop (mean score=4.1) (page s69) (48) | Respondents were also asked about the preferred mode of training or technical assistance. Using a 1–5 rating scale, the most popular option was an onsite workshop (mean score=4.1) (QZ) |
| Respondents were also asked about the preferred mode of training or technical assistance. Using a 1–5 rating scale, the most popular option was an onsite workshop (mean score=4.1), and using a telephone help line (mean score=3.5). (page s69) (48) | The second preferred mode of training or technical assistance was using a telephone help line (mean score=3.5). (QZ) |
| followed by an expert being available to answer questions (mean score=4.0). (page s69) (48) | The third preferred mode of training or technical assistance was an expert being available to answer questions (mean score=4.0).(QZ) |
| help with grant writing (mean score=3.9). (page s69) (48) | The fourth preferred mode of training or technical assistance was help with grant writing (mean score=3.9). (QZ) |
| using a CD-ROM (mean score=3.7). (page s69) (48) | The fifth preferred mode of training or technical assistance was help using a CD-ROM (mean score=3.9). (QZ) |

1. The issue

| Verbatim quantitative findings | Qualitized findings |
| --- | --- |
| Organizational climate - Promoting physical activity is high priority for health department n=32, 65.3%. (page s69) (48) | 65.3% of participants agree or strongly agree that promoting physical activity is high priority for the health department. |

1. The research

| Verbatim quantitative findings | Qualitized findings |
| --- | --- |
| Internal factors hindering its promotion: omission of certain environmental risk factors (n = 5). Pg 4(53). | Out of 28 participants, five have suggested that the omission of certain environmental risk factors hinder the ECAC’s promotion. |
| internal factors hindering its promotion - the presentation of ECAC suggests all 12 messages are equivalent in their importance to the cancer burden (n = 4) Pg 4(53). | Out of 28 participants, four suggested that the presentation of ECAC suggests all 12 messages are equivalent in their importance to the cancer burden and it hinders its promotion. |
| Comprehensiveness of the cancer prevention topics addressed (n = 15) Pg 4 (53). | Out of 28 participants, fifteen cited the comprehensiveness of the cancer prevention topics addressed as a positive internal factor. |
| The robust evidence base underpinning the recommendations (n = 11) Pg 4 (53). | Out of 28 participants, eleven cited the robust evidence base underpinning the recommendations as a positive internal factor. |
| The prominence given to the main modifiable risk factors (n = 5), which have been emphasised consistently in each edition of ECAC (n = 4) Pg 4 (53). | Out of 28 participants, five cited the prominence given to the main modifiable risk factors as a positive internal factor which four participants have noted that they were emphasised consistently in each edition of the ECAC. |

1. The researcher-user relationship – Not reported
2. Dissemination strategies

| Verbatim quantitative findings | Qualitized findings |
| --- | --- |
| Uniformity of audience: When considering internal factors hindering its promotion, promoters most frequently noted the “one-size-fits-all” approach towards addressing the general population (n = 13) Pg 4 (53). | Verbatim finding used for analysis |
| Uniformity of messages: the standardisation of the 12 messages of ECAC was identified as a hindrance (n = 10) Pg 4 (53). | Verbatim finding used for analysis |
| Disadvantages of the ECAC influencing its promotion and dissemination: Several promoters […] felt that the length of the 4th edition (n = 7) and omission of certain environmental risk factors (n = 5) created difficulties in the promotion of the ECAC. Pg 4 (53). | Verbatim finding used for analysis |
| Unaware before taking survey n=30 %=22.2, 95%CI (15.0-29.0).(47) | About 22% of participants have reported that they were not aware of the materials before taking the survey. |
| The clarity of phrasing for each recommendation (n = 14) Pg 4 (53). | Verbatim finding used for analysis |
| The coordination and endorsement of the International Agency for Research on Cancer (IARC) (n = 5) and the European Commission (n = 5) provides important credibility. Pg 4 (53). | Verbatim finding used for analysis |
| The assessment of awareness revealed that 77.8% of respondents were aware of the materials before receiving the survey invitation. The initial source of awareness varied in type: Links to PDF files from the NSPAPPH Website n=51 %=37.8 95%CI (29.0-46.0). (47) | About 38% of participants have reported that they were aware of the materials through links to PDF files from the NSPAPPH [National Society of Physical Activity Practitioners in Public Health] Website before receiving the survey. |
| The top vehicles of awareness were means of dissemination such as: e-mail subscription/list-serve n=48 %=35.6 95%CI (28.0-44.0). (47) | About 48% of participants have reported that they were aware of the materials through e-mail subscription/list-serve before receiving the survey. |
| The top vehicles of awareness were means of dissemination such as: Links to PDF files from the CDC DNPAO Website n= 30 %=22.2 95%CI (15.0-29.0). (47) | About 22% of participants have reported that they were aware of the materials through links to PDF files from the CDC [Centers for Disease Control] DNPAO [Division of Nutrition, Physical Activity and Obesity] Website before receiving the survey. |
| Other forms of information distribution such as: CDC contacts n=23 %=12.6, 95%CI (11.0-23.0). (47) | About 13% of participants have reported that they were aware of the materials through CDC contacts before receiving the survey. |
| Other forms of information distribution such as: State or local health department contacts n=17, %=5.2, 95%CI (7.0-18.0). (47) | About 5% of participants have reported that they were aware of the materials through State or local health department contacts before receiving the survey. |
| Other forms of information distribution such as: Federal agency n=7, %=4.4, 95%CI (1.0-9.0). (47) | About 4% of participants have reported that they were aware of the materials through Federal agency before receiving the survey. |
